# Influences of Linker and Nucleoside for the Helical Self-Assembly of Perylene Along DNA Templates

**DOI:** 10.3389/fchem.2019.00659

**Published:** 2019-10-22

**Authors:** Yannic Fritz, Hans-Achim Wagenknecht

**Affiliations:** Karlsruhe Institute of Technology, Institute of Organic Chemistry, Karlsruhe, Germany

**Keywords:** DNA, chromophor, assembly, template, fluorescence

## Abstract

Six different conjugates of perylene with 2′-deoxyuridine and with 2-amino-2′-deoxyadenosine were synthesized and applied for DNA-templated assembly in aqueous buffer solutions. They differ by the linkers ethynylene, phenylene, and phenylene–ethynylene between nucleoside and chromophore. The nucleosides were investigated as monomers in CHCl_3_ and dimethyl sulfoxide by optical spectroscopy. The properties of the four phenylene-linked conjugates are similar to that of perylene as reference because these linkers separate both aromatic parts. The ethynylene linker electronically couples the chromophore with the respective nucleoside and thus red shifts the absorbance. The DNA-templated assembly properties were elucidated by mixing the templates in aqueous buffer with the perylene–nucleoside conjugates from a dimethyl sulfoxide stock solution. Specific binding of the nucleosides was probed by comparing the results with dA_20_ and T_20_ as single-stranded DNA templates. Our studies reveal the structural parameters that are important for the DNA-templated assembly of perylenes. First, perylene-2′-deoxyuridine conjugates do not form DNA-templated helical assemblies, regardless of the choice of linker. Second, the ethynylene linker is crucial for successful DNA-templated chromophore assemblies of perylene-2-amino-2′-deoxyadenosine conjugates. Third, in contrast, the phenylene linker inhibits self-assembly along single-stranded DNA templates. In conclusion, the 2-amino-2′-deoxyadenosin in combination with the ethynylene linker provides the best structural feature for specific and helical DNA-templated assembly of perylenes. This result is important for the design of future DNA-based supramolecular architectures with chromophores, in particular DNA-based light-harvesting systems and DNA systems for emitting or sensing circularly polarized luminescence.

**Graphical Abstract:**
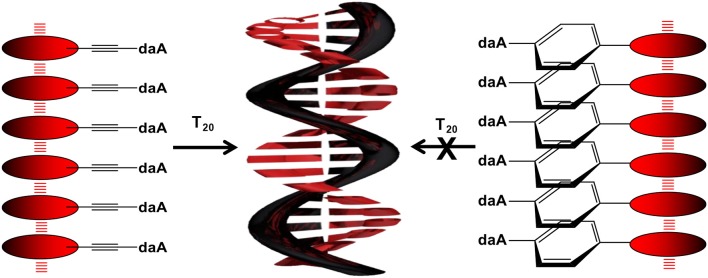
Illustration of the DNA-templated assembly of **Pe–Et–daA** and **Pe–Ph–daA**.

## Introduction

Supramolecular chemistry and supramolecular polymerization summarize the efforts to self-organize molecules through interactions in a precise and controllable way. Probably more than any other class of compounds, DNA provides a rather unexplored and groundbreaking new access to this challenge via bottom–up, programmed, and hierarchically ordered assembly of molecules, in particular organic chromophores, into designed supramolecular DNA architectures. The last two centuries have shown that—beyond their pure biological purpose—nucleic acids are able to serve as unique structural basis to create nanometer-sized two- and three-dimensional objects. Representatively, the DNA nanostructures of Seeman (1982) and Kallenbach et al. (1983) and the DNA origami's by Rothemund (2006) are mentioned here. Since then, “DNA nanotechnology” has been established as the approach that applies self-assembly of DNA single strands to form molecular architectures in a highly organized manner (Yakovchuk et al., 2006; Lubrich et al., 2008; Wang et al., 2009; Chen et al., 2019). The sequence-defined recognition by canonical base pairing in DNA in combination with a perfectly coplanar stacking distance of 3.4 Å and a helical chirality should yield supramolecular architectures (Burge et al., 2006). Such architectures are difficult to be achieved by simple organic-chemical building blocks without DNA, neither covalently (polymers) nor non-covalently (supramolecular polymers). In a bottom–up approach, we follow herein the basic principle of chemistry research that “structure determines properties.” Therefore, the controlled assembly of organic chromophores in supramolecular architectures based on nucleic acids holds the key potential for future functional materials with well-defined photochemical properties. In particular, the helical twist between the chromophores, which is induced by the DNA scaffold, controls electron transfer and energy transfer processes and thereby reduces the self-quenching that is typically observed in chromophore aggregates (Asanuma et al., 2003; Teo et al., 2009; Dutta et al., 2011; Kato et al., 2013; Li et al., 2013; Probst et al., 2014; Ishutkina et al., 2018).

In 2009, Kumar and Duff presented one of the first DNA-based light harvesting systems (Kumar and Duff, 2009). In the same year, our group published a temperature-controlled white-light-emitting DNA by covalent incorporation of pyrene- and Nile-red-modified nucleosides (Varghese and Wagenknecht, 2009). The combination with a perylene–nucleoside yielded a light-harvesting antenna, and thereby, any excitation in the range between 350 and 600 nm efficiently resulted in a charge-separated state (Ensslen et al., 2015a). Two years later, the influence of the template strand length on the energy transfer of attached naphthalene nucleosides was investigated by the group of Stevens (Ruiz-Carretero et al., 2011; Stevens et al., 2011). Balaz et al. created nanoassemblies with porphyrine-linked 2-aminoadenines and oligothymidine as DNA templates, which showed an adjustable helicity depending on temperature gradients (Sargsyan et al., 2014). With pyrene and Nile red attached to different nucleosides, a sequence-specific self-assembly along a template DNA strand was accomplished in 2018 (Hofsass et al., 2018). Pyrene was linked via ethynylene bridge to 2-amino-2′-deoxyadenosine (daA) and Nile red to 2′-deoxyuridine (dU), which are complementary to thymine (T) and adenine (A), respectively, as recognition units of the template. Moreover, fullerene–DNA–chromophore assemblies were integrated as photoactive layers in solar cells that showed charge carrier generation in the spectral regime of all three components (Ensslen et al., 2016). These recent examples demonstrate the significance and potential of supramolecular DNA architectures.

**Figure 1:**
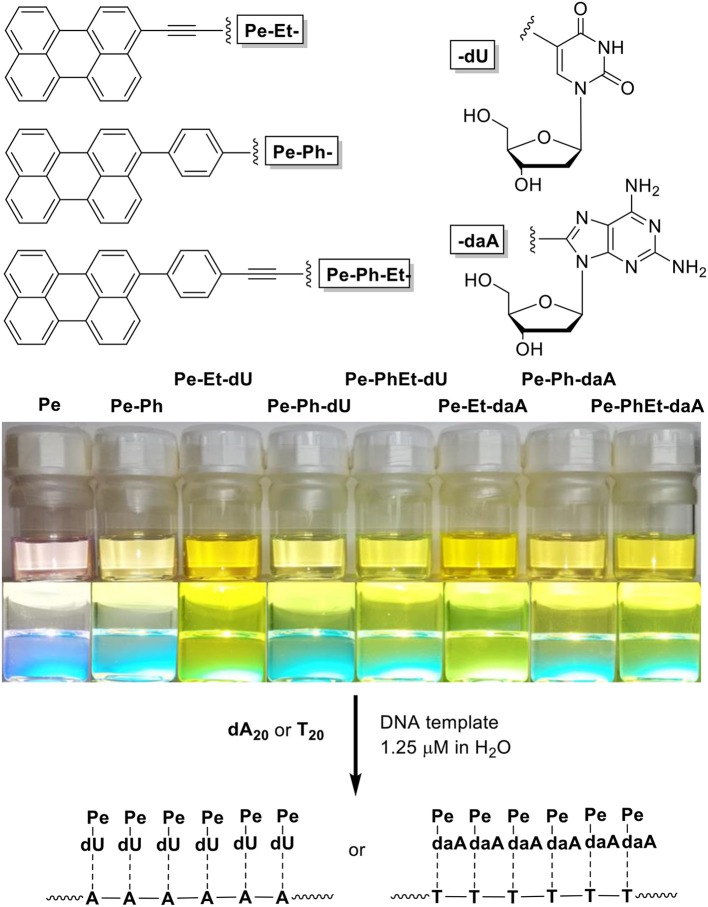
**(Top)** Structure of the nucleoside conjugates **Pe–Et–dU**, **Pe–Ph–dU**, **Pe–Ph–Et–dU**, and **Pe–Et–daA**, **Pe–Ph–daA**, **Pe–Ph–Et–daA**. **(Bottom)** Image of solutions of perylene (**Pe**), 3-phenylperylene (**PePh**), and the nucleoside conjugates in CHCl_3_/dimethyl sulfoxide (DMSO) = 4:1 (100 μM) and image of their fluorescence during excitation by a UV handheld lamp.

To get a closer insight into the influence of linker and nucleoside on the optical and self-assembly properties with DNA, six different perylene-conjugated nucleosides (Figure 1) are presented herein that differ (i) by the linker between the chromophore and the nucleoside, including ethynylene (Et), phenylene (Ph), and the combined phenylene–ethynylene (PhEt) linkers, and (ii) by the nucleoside, either dU that recognizes A in the DNA template, or daA that recognizes T in the DNA template. The Et linker yields a coplanar orientation of nucleoside and perylene parts in the conjugates, whereas the Ph linker introduces a rotational twist between the two parts. The PhEt linker combines the two extreme geometries and could potentially serve as compromise. We study the self-assembly of these nucleoside conjugates and the influence of the corresponding single-stranded DNA templates oligothymidine (**T**_**20**_) and oligo-2′-deoxyadenosine (**dA**_**20**_) in comparison by methods of optical spectroscopy to characterize their optical and chiroptical properties.

## Materials and Methods

All chemicals used for synthesis had at least the purification grade “for synthesis.” Solvents used in synthesis, optical spectroscopy or analysis had the grade “HPLC” or “pro analysis.” Water was deionized and ultra-filtrated by a Millipore Direct 8/16 from MERCK MILLIPORE. Unmodified DNA strands were bought from METABION, which were already HPLC-purified and lyophilized. The DNA was dissolved in water and concentrations were determined spectrometric with a NANODROP ND-100 spectrophotometer. All Pd-catalyzed reactions were performed under exclusion of oxygen and water (except for Suzuki-couplings, which used water as reagent). Reaction mixtures were treated by freezepump-thaw in three cycles before adding the catalyst or degassed with argon. Some reactions were performed in sealed glass vials (10 mL or 20 mL), which resulted in significantly higher yield compared to common round bottom flasks. The purity of all products were determined by NMR-spectroscopy and high-resolution mass spectrometry. NMR spectra were recorded on a BRUKER Advance 500 (500 MHz, 1H-NMR; 126 MHz, 13C-NMR). Chemical shifts were reported in parts per million (ppm), relative to the standard tetramethylsilane (δ = 0.00 ppm) and the spectrum was calibrated against the 1 H residues of the deuterated solvents. Due to the bad solubility of most products, the use of deuterated pyridine was necessary. The mass-spectrometry was performed on a THERMOFISHER Scientientific Q Exactive (Orbitrap) by electron spray ionization (ESI) and reported in mass/charge (m/z). In case of daA-containing products, the protonated species was mostly found. For all spectroscopic experiments semi-micro quartz glass cuvettes from STARNA (width 10 mm, volume 1.4 mL) were used and all spectra were recorded at 20°C. Absorption spectra were recorded on a Lambda 750 from PERKIN ELMER with a PTP-6+6 Peltier System. Circular dichroism was measured with a JASCO J-810 Spectropolarimeter and the peltier-element PTC-423S (100 nm/min, 4 accumulations). Fluorescence was recorded on a Fluoromax-4 from HORIBA SCIENTIFIC with an AC 200 thermostat from THERMO SCIENTIFIC. All samples were excited at 420 nm and the spectra were divided by the absorbance at 420 nm for comparison. Absolute fluorescence quantum yields were determined with a Quantaurus QY C11347 from HAMAMATSU (measured in different concentrations between 40 and 60 μM and averaged). Self-assembly experiments were prepared as follows: Chromophore 45 mM, DNA template 1.5 μM, phosphate-buffer 10 mM (pH 7.0), 250 mM NaCl. Due to solubility issues, the chromophores were added from a 1 mM stock solution in DMSO, which causes a DMSO content of 4.5%. For CD and fluorescence experiments the Chromophore was added shortly before the measurement, to minimize the influence of precipitation.

## Results and Discussion

**Figure 2:**
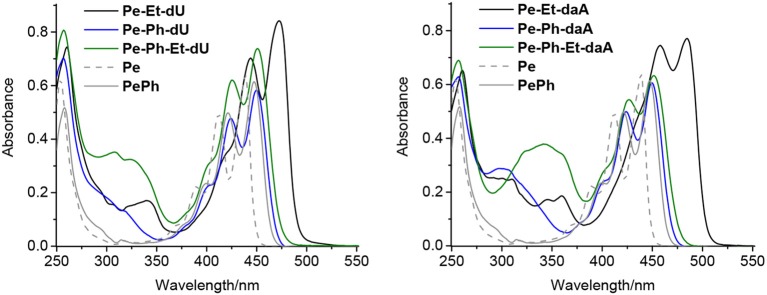
Ultraviolet–visible (UV/vis) absorption spectra of perylene (**Pe**), 1-phenylperylene (**PePh**), and the perylene–nucleosides with dU **(Left)** and with daA **(Right)**, each 20 μM in CHCl_3_ [2% dimethyl sulfoxide (DMSO)].

All nucleoside conjugates were synthesized as described in the Supporting Information. The key steps are Pd-catalyzed Sonogashira and Suzuki couplings between the halogenated nucleoside precursors and the perylene derivatives. We expected that the linker between chromophore and nucleoside has a strong impact on the photophysical properties. In principal, the ethynylene group connects the perylene chromophore with the nucleosides in a coplanar orientation and thereby yields π-conjugation between them, whereas the phenylene group electronically isolates the π-systems by rotation. Indeed, this structural influence is clearly observable by the ultraviolet–visible (UV/vis) absorbance (Figure 2): The characteristic perylene absorbance between 400 and 520 nm is nearly identical for all Ph- and Ph–Et-linked conjugates compared to 1-phenyl-perylene as reference (Kawasumi et al., 2013). In particular, both **Pe–Ph–dU** and **Pe–Ph–daA** show only very little absorbance differences to the reference, which is an advantage of the Ph linker that efficiently interrupts the π-conjugation with the nucleoside. Only the Et-linked conjugates **Pe–Et–dU** and **Pe–Et–daA** display an absorbance that is red shifted by 23 and 35 nm, respectively. This shows the π-conjugating effect of the Et linker.

In order to study the effect of the nucleosides and the linkers on the fluorescence, the spectra and quantum yields were recorded (Table 1 and Figure S1). In CHCl_3_ as solvent [with 6% dimethyl sulfoxide (DMSO) to ensure solubility], the four Ph- and Ph–Et-conjugated nucleosides yield higher quantum yields (77–84% for dU and 83% for daA conjugates) than **Pe–Et–dU** and **Pe–Et–daA** (58 and 78%, respectively). These results agree with the electronically separating character of the Ph linker, too. In contrast, the Et group enlarges the π-system and thus lowers the quantum yields. In conclusion, these results show the expected tuning of the optical properties of the chromophore–nucleoside conjugates by the choice of linker. Surprisingly, the emissions of the daA-modified chromophores are nearly quantitatively quenched in pure DMSO, while the emissions of the dU-modified ones give quantum yields from 76 to 96%. These striking fluorescence quantum yield differences in DMSO become plausible by photoinduced electron transfer in the daA conjugates that occurs only in the highly polar solvent DMSO. Based on redox potentials from literature, daA can be more easily oxidized (*E*_ox_ = 1.1 V vs. NHE in water) (Stockert et al., 2002) than dU (*E*_ox_ = 1.34 V) (Faraggi et al., 1996). Using the reduction potential for perylene of *E*_red_ = −1.91 V together with *E*_00_ = 2.8 eV (Rossetti et al., 2005), the Rehm–Weller equation for the driving forces Δ*G* = *E*_ox_ – *E*_red_ – *E*_00_ + *E*_C_ (Coulomb energy *E*_c_) gives a small Δ*G* of −0.3 eV for the daA conjugates in favor of a photoinduced charge transfer, but a negligible Δ*G* for the dU conjugates (without consideration of *E*_C_) that makes the photoinduced charge transfer very unlikely in the latter conjugates. Obviously, the different polarity of the solvents DMSO and CHCl_3_ influence the Coulomb energy in the charge-separate state such that only in the highly polar solvent DMSO the photoinduced electron transfer in the daA conjugates is observable. In DMSO, the charge-separated state is stabilized and thereby the Coulomb energy is reduced.

**Table 1:** Fluorescence quantum yields of perylene (**Pe**), 1-phenylperylene (**PePh**), and the perylene–nucleosides in DMSO and CHCl_3_ with 6% DMSO (average of 40, 50, and 60 μM), λ_exc_ = 420 nm.

**Nucleoside**	****Φ_F_** in DMSO**	****Φ_F_** in CHCl_**3**_ (6% DMSO)**
**Pe–Et–dU**	76 ± 0.97%	58 ± 1.1%
**Pe–Ph–dU**	96 ± 1.9%	84 ± 0.16%
**Pe–Ph–Et–dU**	90 ± 1.6%	77 ± 0.45%
**Pe–Et–daA**	6.7 ± 0.21%	78 ± 0.66%
**Pe–Ph–daA**	2.8 ± 0.08%	83 ± 0.21%
**Pe–Ph–Et–daA**	5.5 ± 0.16%	83 ± 0.21%
**Pe**	~100%	98 ± 0.60%
**PePh**	~100%	~100%

**Figure 3:**
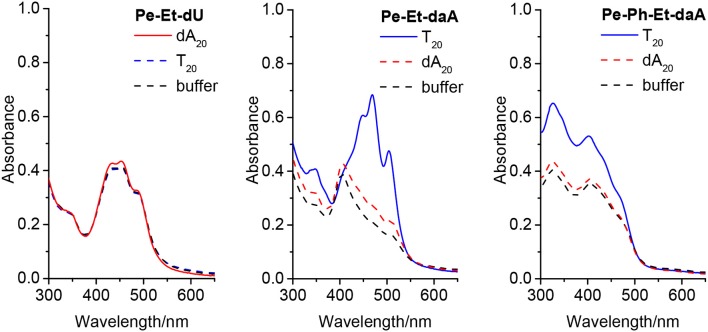
Ultraviolet–visible (UV/vis) absorbance of **Pe–Et–dU (Left)**, **Pe–Et–daA (Middle)**, and **Pe–Ph–Et-daA (Right)**, each 45 μM in aqueous buffer (10 mM sodium phosphate, pH 7, 250 mM NaCl, with 4.5% DMSO) without and with DNA templates **T**_**20**_ and **dA**_**20**_ (1.5 μM), respectively.

To examine the self-assembling ability of the nucleoside conjugates, experiments with **T**_**20**_ and **dA**_**20**_, respectively, as single-stranded DNA templates were performed. The chromophore conjugates were characterized by their UV/vis absorbance, fluorescence, and circular dichroism in aqueous buffer at pH 7 to elucidate (i) their self-assembling properties and, more importantly (ii) the potential influence of the single-stranded DNA templates. Specific binding of the nucleoside conjugates was elucidated by comparing the results with **dA**_**20**_ and **T**_**20**_ as templates. Previous work in our group revealed that the assembly of nucleosides along DNA templates succeeded at room temperature with no annealing (Hofsass et al., 2018). We kept this method for the studies herein. The shapes of all absorption spectra in H_2_O differ from those of the monomer reference spectra in DMSO. First, the absorbance of the assemblies of **Pe–Et–dU** (Figure 3, left) and **Pe–Et–daA** (Figure 3, middle) along the DNA templates were compared to DNA-free samples in aqueous buffer (with 4.5% DMSO from the perylene-nucleoside stock solutions). In case of **Pe–Et–dU**, the perylene-typical fine structure is significantly broadened, indicating self-assembly of the chromophores. There is only little influence of the complementary DNA template **dA**_**20**_, if at all, by a slightly increased absorbance compared to the non-complementary template **T**_**20**_ and compared to the absence of any DNA, too. For **Pe–Et–daA** in the presence of the complementary template **T**_**20**_, however, the perylene-typical fine structure is maintained but with an altered ratio of the included absorption bands. With the non-complementary template **dA**_**20**_ and without any DNA, this fine structure is completely lost, and the absorbance is significantly blue shifted to a broad absorbance from 350 to 550 nm with a maximum at 408 nm. Since this absorbance is similar to that without the template, we assign it to the non-templated self-assembly of **Pe–Et–daA**. The influence of the complementary DNA template **T**_**20**_ is significant and serves as first indication for a base pairing of the **Pe–Et–daA** monomers along this template and thus the formation of an ordered and helical assembly (*vide infra*). It is important to mention here that daA is, in principal, a modified 2′-deoxyadenosine with a second amino group to enhance the binding selectivity by a potential third hydrogen bond to thymidines as recognition unit in the DNA template **T**_**20**_ (Hofsass et al., 2018). The lacking third hydrogen bond between **Pe–Et–dU** and the DNA templates probably accounts for its lacking selectivity between **dA**_**20**_ and **T**_**20**_ (and without template). The UV/vis absorbances of the remaining four nucleosides with Ph- and Ph–Et linkers in water look all similar and do not allow to elucidate any influence of the single-stranded DNA templates on the formation of these chromophore assemblies (Figure 3, right and Figure S3). This indicates that the Ph linkers are not all suitable for DNA-templated assembly of perylenes. A careful look on the absorbances reveals, however, that only **Pe–Ph–Et–daA** shows an enhanced absorbance at 408 nm in the presence of the complementary template **T**_**20**_ compared to **dA**_**20**_ and to pure buffer (Figure 3, right), indicating that there might be a small structural influence of the DNA on this particular chromophore assembly.

**Figure 4:**
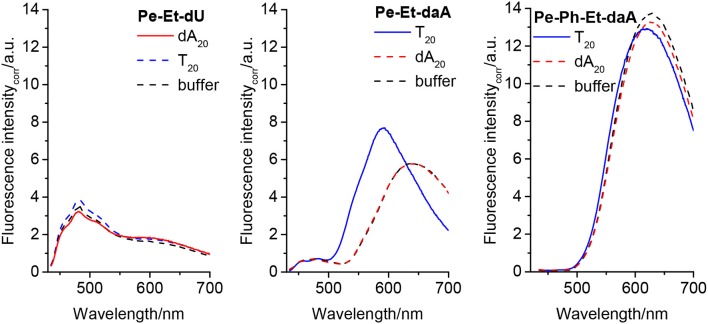
Fluorescence of **Pe–Et–dU (Left)**, **Pe–Et–daA (Middle)**, and **Pe–Et–daA (Right)**, each 45 μM in aqueous buffer (10 mM sodium phosphate, pH 7, 250 mM NaCl, with 4.5% DMSO) without and with DNA templates **T**_**20**_ and **dA**_**20**_ (1.5 μM), respectively. Excitation at 420 nm. The fluorescence intensity was corrected by the absorbance at 420 nm.

The fluorescence further supports the elucidation of structural prerequisites for the assembly of perylene–nucleoside conjugates along single-stranded DNA templates that were indicated by their UV/vis absorbance. In all non-templated samples, an unstructured and broad fluorescence was observed at ~600–650 nm, which we assign to perylene excimers as a result of the self-assembly of the perylene–nucleoside conjugates in water. In particular, both **Pe–Et–dU** and **Pe–Et–daA** without DNA templates show this unstructured and broad fluorescence at 600 and 650 nm, respectively (Figure 4, left and middle). If we assume a coplanar arrangement by π-π stacking between the perylene monomers and Watson–Crick or any similar base pairing between the nucleoside conjugates and the templates, a helical twist is introduced by the DNA to the perylene assembly in water. According to our previous results with pyrene conjugates, such helicity typically reduces excimer-like fluorescence (Trifonov et al., 2005). In fact, the assembly in the presence of the template **T**_**20**_ significantly changes the fluorescence readout of **Pe–Et–daA**. This sample shows mainly a blue-shifted and enhanced fluorescence at 590 nm, which we assign to the ordered and presumably helical assembly of these chromophores. This assignment supports the considerably different absorbance of **Pe–Et–daA** with **T**_**20**_ as described above. The assembly of **Pe–Et–dU** shows only fluorescence between 450 and 550 nm and only little excimer fluorescence intensity. The lacking influence of neither **dA**_**20**_ nor **T**_**20**_ as complementary or non-complementary templates on the fluorescence readout implies that **Pe–Et–dU** forms a stable non-templated self-assembly. This stands in contrast to **Pe–Et–daA** for which only the complementary DNA template **T**_**20**_ orders the chromophore assembly in such a way that the fluorescence is blue shifted. In contrast to the two Et-linked nucleosides, the fluorescence of **Pe–Ph–dU**, **Pe–Ph–daA**, and **Pe–Ph–Et–dU** show only excimer-like fluorescence of the non-templated and self-assembled perylene conjugates at 650 nm, which is not significantly altered by the DNA templates (Figure S2). Similar to the observations by UV/vis absorption spectroscopy, the DNA templates have no significant effect on the self-assembly of the Pe–Ph conjugates. Obviously, the twist introduced by the Ph in these nucleoside conjugates disturbs the coplanar orientation of nucleoside and perylene and thereby inhibits the templated assembly along the single-stranded DNA. Typical DNA double helix has a stacking distance of 0.34 nm between two base pairs, whereas the diameter of Ph is 0.43 nm; a rotational twist in the Ph-linked conjugates cannot be accepted by this type of assembly. A careful look onto the fluorescence of **Pe–Ph–Et–daA** reveals a small blue shift from 628 nm (with the non-complementary template **dA**_**20**_ and without any template) to 618 nm with the complementary template **T**_**20**_ (Figure 4, right) and thereby supports the small influence of this DNA template on this particular chromophore assembly similarly to the previously discussed UV/vis absorbance.

**Figure 5:**
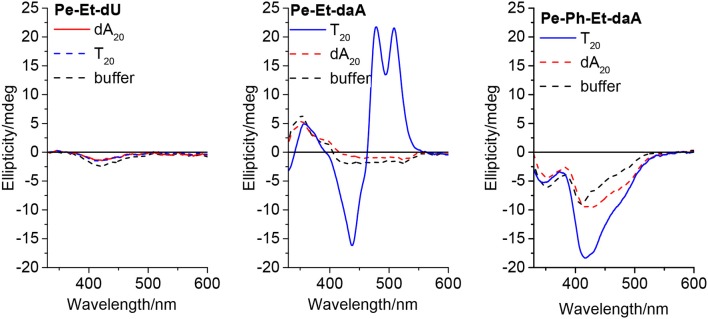
Circular dichroism of **Pe–Et–dU (Left)**, **Pe–Et–daA (Middle)**, and **Pe–Ph–Et–daA (Right)**, each 45 μM in aqueous buffer (10 mM sodium phosphate, pH 7, 250 mM NaCl, with 4.5% DMSO) without and with DNA templates **T**_**20**_ and **dA**_**20**_ (1.5 μM), respectively.

To get a closer look into the possible helical orientations of the perylene–nucleoside conjugates introduced by the single-stranded DNA template, circular dichroism (CD) was recorded for all samples. We do not interpret the CD in the range between 250 and 350 nm because the perylene–nucleoside absorbance overlaps with the template absorbance. All nucleoside conjugates without DNA template display only small chiroptical activity in DMSO but no sign for ordered helical arrangements (Figure S4). These signals mainly arise from the chromophores connected to the chiral β-d-ribofuranoside residue and in particular in DMSO as good solvent for both parts of the nucleoside conjugates, polar and non-polar. The CD spectra of **Pe–Et–dU** with **dA**_**20**_, with **T**_**20**_ and without any DNA template are similar to those of the monomers in DMSO (Figure 5, left). This result further supports our explanation that the stable, non-templated self-assemblies of **Pe–Et–dU** are not chiral and they are not significantly influenced by the DNA template. The intrinsic self-stacking of the perylenes overrules any template effect. Similarly, small CD signals are observed in the samples with **Pe–Ph–dU**, **Pe–Ph–daA**, and **Pe–Ph–Et–dU** (Figure S5). This makes conclusively clear that two potential hydrogen bonds, which are provided by the dU unit, are not sufficient for effective and specific assembly of perylenes along DNA templates. In contrast, both **Pe–Et–daA** and **Pe–PhEt–daA** show strong chiroptical signals, which are enhanced in presence of **T**_**20**_. The CD of **Pe–Et–daA** with **T**_**20**_ (Figure 5, middle) displays a clearly excitonically coupled signal in the perylene absorbance range between 350 and 550 nm by the combination of a negative Cotton effect followed by a positive Cotton effect. This indicates a left-handed chirality in these DNA-based assemblies with coplanarily stacked **Pe–Et–daA** chromophores. We previously observed this chirality for DNA-templated assemblies with pyrene- and Nile-red-modified nucleosides (Ensslen et al., 2015b; Hofsass et al., 2018). Even stronger CD signals were observed for the templated assemblies of **Pe–Ph–Et–daA** with **T**_**20**_ (Figure 5, right) but not for **Pe–Ph–Et–dU** with **dA**_**20**_ (Figure S5). Obviously, the Ph–Et linker still allows the chromophore assembly along **T**_**20**_, but the perylene orientation differs from that of the clearly excitonically couples and coplanarily stacked **Pe–Et–daA** assembly with **T**_**20**_ because the CD shows only a negative Cotton effect. Overall, there are two major results with respect to the structural parameters: (i) The structural influence of the DNA template is observed only with the daA conjugates but not with the dU conjugates, and (ii) while the Et-linked chromophores with daA selectively assemble along the complementary template strand with left-handed chirality, the Ph-linked chromophores do not form templated water-soluble assemblies. Only the conjugate of daA with both linkers (Ph–Et) interact with the DNA templates in a different type of chiral assembly.

## Conclusions

Six different conjugates of perylene with dU and with daA were synthesized and applied for DNA-templated assembly in aqueous solutions. The perylene conjugates differ by the linkers between the perylene chromophore and by the aromatic nucleoside heterocycle. The photophysical properties of the nucleoside monomers and their self-assemblies were investigated by optical spectroscopy methods. The Ph linker as well as the combined Ph–Et linker separate both aromatic parts. Accordingly, the optical properties of the Pe–Ph and Pe–Ph–Et conjugates with dU and daA are similar to that of perylene and 1-phenylperylene as reference chromophores. In comparison, the Et linker electronically couples the chromophores and thus red shifts the absorbance. The DNA assemblies were formed by mixing the templates in water with the perylene–nucleoside conjugates from a DMSO stock solution. Important structural parameters for the DNA-templated assembly of perylenes were elucidated: (i) Perylene–dU conjugates do form stable, non-templated assemblies and overrule the DNA template effect. dU can base pair only by two hydrogen bonds with the nucleotides in the DNA template, whereas daA provides the donors and acceptor for three hydrogen bonds. Although we do not provide direct experimental evidenced for base pairing via hydrogen bonding similar to Watson–Crick base pairing, this difference seems to be striking in particular for the comparison of the assemblies with **Pe–Et–dU** vs. with **Pe–Et–daA**. (ii) The Et linker is required for successful DNA-templated chromophore assemblies of the conjugate with daA. It orientates both aromatic parts in a coplanar orientation. Obviously, this facilitates the formation of a helical DNA-templated assembly with presumably π-π-stacked coplanarily arranged perylenes, which was evidenced in particular by the assemblies of **Pe–Et–daA** in comparison with **Pe–Ph–daA**. (iii) The Ph linker twists both aromatic parts and thus completely inhibits self-assembly along single-stranded DNA templates in aqueous solutions. Accordingly, the conjugate **Pe–Et–daA** shows the strongest selectivity to the complementary DNA templates, while the Ph-linked one did not form templated assemblies. The assembly of **Pe–Et–daA** with **T**_**20**_ shows a left-handed chirality and excitonic coupling. The conjugate **Pe–Ph–Et–daA** with both linkers shows also a structural influence by **T**_**20**_ as DNA template in particular according to the chiroptical properties; however, the structure of this perylene assembly significantly differs from the coplanar and excitonically coupled **Py–Et–daA** assembly. Overall, the daA nucleoside in combination with the Et linker provides the two structural prerequisites for specific and helical DNA-templated assembly that were identified by our study. This result is in agreement with other DNA-templated chromophore arrangements by us and others (Stevens et al., 2011; Sargsyan et al., 2014; Hofsass et al., 2018). Such investigations are important for the design of DNA-based supramolecular architectures with chromophores, in particular DNA-based light-harvesting systems and DNA architectures for emitting or sensing circularly polarized luminescence.

## Data Availability Statement

All datasets generated for this study are included in the manuscript/Supplementary Files.

## Author Contributions

YF did all experiments and wrote parts of the manuscript. H-AW supervised the research and wrote the manuscript.

### Conflict of Interest

The authors declare that the research was conducted in the absence of any commercial or financial relationships that could be construed as a potential conflict of interest.

## Experimental part

All experimental procedures are described in the Supporting Information.
