# Supplementary material for: Influences of Linker and Nucleoside for the Helical Self-Assembly of Perylene Along DNA Templates
Source: Front Chem. 2019 Oct 22;7:659. doi: 10.3389/fchem.2019.00659 (PMC6817502; doi:10.3389/fchem.2019.00659)
Supplement: Supplementary file 1 [file Data_Sheet_1.pdf]

Influences of Linker and Nucleoside for the Helical Self-assembly of Perylene along DNA Templates

Yannic Fritz, Hans-Achim Wagenknecht*

Supporting Information

Karlsruhe Institute of Technology (KIT), Institute of Organic Chemistry, Fritz-Haber-Weg 6, 76131 Karlsruhe, Germany

*Corresponding author

E-mail: wagenknecht@kit.edu

Content

Materials and Methods 2

Synthesis and characterization 4

Additional Spectra 40

Emission spectra 40

UV/vis-absorption spectra 42

CD-spectra 43

References 44

Materials and Methods

All chemicals used for synthesis had at least the purification grade "for synthesis". Solvents used in synthesis, optical spectroscopy or analysis had the grade "HPLC" or "pro analysi". Water was deionized and ultra-filtrated by a *Millipore Direct 8/16* from MERCK MILLIPORE. Unmodified DNA strands were bought from METABION, which were already HPLC-purified and lyophilized. The DNA was dissolved in water and concentrations were determined spectrometric with a NANODROP ND-100 spectrophotometer.

All Pd-catalyzed reactions were performed under exclusion of oxygen and water (except for Suzuki-couplings, which used water as reagent). Reaction mixtures were treated by freeze-pump-thaw in three cycles before adding the catalyst or degassed with argon. Some reactions were performed in sealed glass vials (10 mL or 20 mL), which resulted in significantly higher yield compared to common round bottom flasks.

The purity of all products were determined by NMR-spectroscopy and high-resolution mass-spectrometry. NMR spectra were recorded on a BRUKER *Advance 500* (500 MHz, ^{1}H-NMR; 126 MHz, ^{13}C-NMR). Chemical shifts were reported in parts per million (ppm), relative to the standard tetramethylsilane (δ = 0.00 ppm) and the spectrum was calibrated against the ^{1}H-residues of the deuterated solvents. Due to the bad solubility of most products, the use of deuterated pyridine was necessary. The mass-spectrometry was performed on a THERMOFISHER *Scientientific Q Exactive* (*Orbitrap*) by electron spray ionization (ESI) and reported in mass/charge (m/z). In case of **daA**-containing products, the protonated species was mostly found.

For all spectroscopic experiments semi-micro quartz glass cuvettes from STARNA (width 10 mm, volume 1.4 mL) were used and all spectra were recorded at 20 °C. absorptionspectra were recorded on a *Lambda 750* from PERKIN ELMER with a *PTP-6+6 Peltier System*. Circular dichroism was measured with a JASCO *J-810 Spectropolarimeter* and the peltier-element *PTC-423S* (100 nm/min, 4 accumulations). Fluorescence was recorded on a *Fluoromax-4* from

HORIBA SCIENTIFIC with an *AC 200* thermostat from THERMO SCIENTIFIC. All samples were excited at 420 nm and the spectra were divided by the absorbance at 420 nm for comparison. Absolute fluorescence quantum yields were determined with a *Quantaurus QY C11347* from HAMAMATSU (measured in different concentrations between 40 µM and 60 µM and averaged).

Self-assembly experiments were prepared as follows: Chromophore 45 mM, DNA template 1.5 µM, phosphate-buffer 10 mM (pH 7.0), 250 mM NaCl. Due to solubility issues, the chromophores were added from a 1 mM stock solution in DMSO, which causes a DMSO content of 4.5 %. For CD and fluorescence experiments the Chromophore was added shortly before the measurement, to minimize the influence of precipitation.

Synthesis and characterization

The precursors **2** - **5** were synthesized according to the literature.(Riedl et al., 2012; Yamaji et al., 2012; Okamoto et al., 2016; Hayashi and Inouye, 2017) The commercially available Nucleoside **6** was protected by the method of OKAMOTO *et al*.(Okamoto et al., 2005) The herein presented synthesis of **Pe-dU** deviates from the known literature.(Andronova et al., 2003)

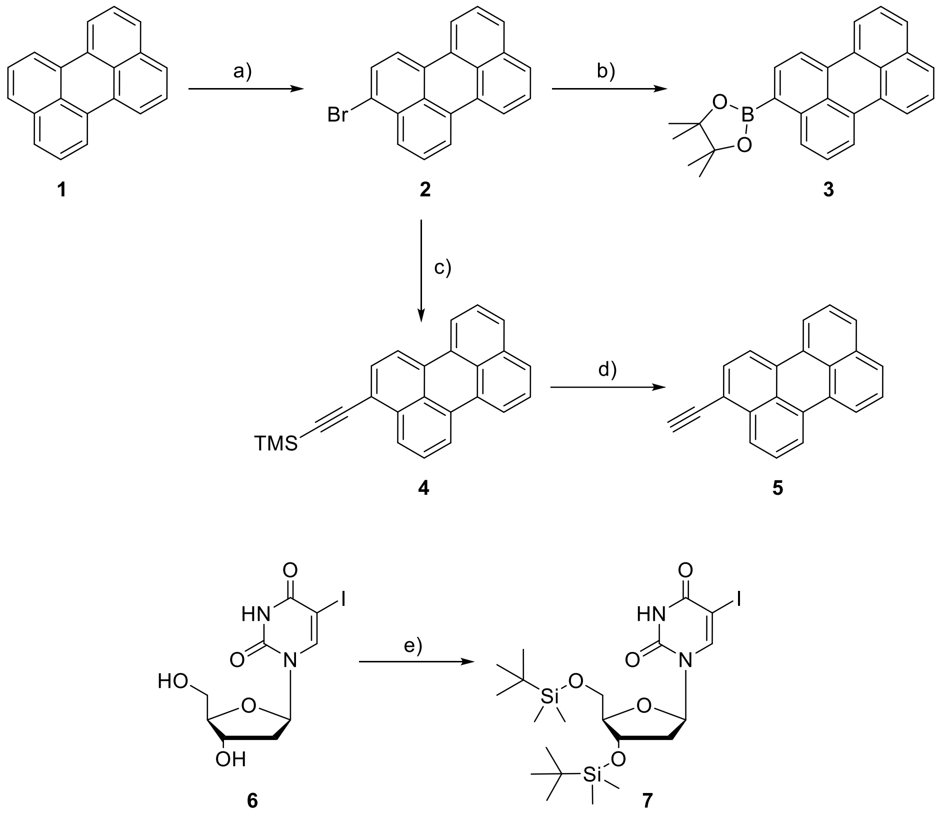

***Scheme S1**: Synthesis of **3** and **5**: a) NBS, THF, r. t., 16 h. b) B_2Pin_2, KOAc, $PdCl_2$(dppf), dioxane, 70 °C, 17 h, 76%. c) TMS-acetylene, $PdCl_2(PPh_3)_2$, CuI, NEt_3, DMF, 80 °C, 16 h, 68%. d) K_2CO_3, MeOH, r. t., 60 h, quant. e) TBDMS-Cl, imidazole, DMF, r. t., 16 h,72%.*

5-(Perylene-3-ylethynyl)-2‘-deoxyuridine (Pe-Et-dU)

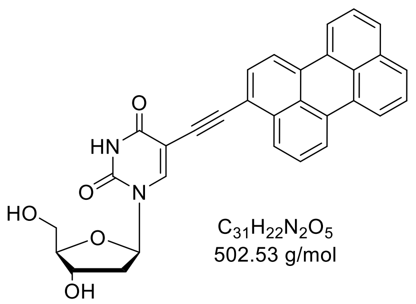

$C_{31}H_{22}N_2O_5$
502.53 g/mol

Pe-Et-dU

5 (195 mg, 706 µmol, 1.00 eq.) was dissolved in dry DMF (20 mL). Tetrakis(triphenylphosphine)palladium (163 mg, 141 µmol, 0.20 eq.), CuI (25 mg, 141 µmol, 0.20 eq.) and NEt_3 (20 mL) were subsequently added and degassed. After addition of **6** (250 mg, 706 µmol, 1.00 eq.), the mixture was stirred at 50 °C overnight. The solvent was removed under reduced pressure and the crude product was purified by silica gel column chromatography (CH_2Cl_2:MeOH 100:1 - 25:1). The product was isolated as an orange solid in a yield of 52% (186 mg, 370 µmol).

TLC R_f(EtOAc) = 0.36

^{1}H NMR (300 MHz, Pyr-d_5): δ (ppm) = 13.97 (s, $1H_{imido}$), 9.31 (s, 1H, 6_{dU}), 8.85 (d, *J* = 8.3 Hz, $1H_{arom.}$), 8.34 – 8.14 (m, $4H_{arom.}$), 7.74 (dd, *J* = 8.1, 2.3 Hz, $2H_{arom.}$), 7.65 (d, *J* = 8.0 Hz, $1H_{arom.}$), 7.60 – 7.53 (m, $1H_{arom.}$), 7.53 – 7.45 (m, $2H_{arom.}$), 7.00 (t, *J* = 6.1 Hz, 1H, $1'_{dU}$), 5.14 (q, *J* = 5.0 Hz, 1H, $3'_{dU}$), 4.55 (d, *J* = 3.1 Hz, 1H, $4'_{dU}$), 4.38 – 4.32 (m, 1H, $5'_{dU}$), 4.24 (dd, *J* = 11.8, 2.8 Hz, 1H, $5'_{dU}$), 2.86 – 2.77 (m, 2H, $2'_{dU}$).

^{13}C NMR (126 MHz, Pyr-d_5): δ (ppm) = 163.4 (C_q), 151.4 (C_q), 144.7 (6_{dU}), 135.6 (C_q), 135.5 (C_q), 132.5 (C_q), 132.3 (C_q), 131.8 (C_q), 131.5 (C_q), 131.3 ($CH_{arom.}$), 129.3 ($CH_{arom.}$, C_q), 129.1 ($CH_{arom.}$), 128.5 ($CH_{arom.}$), 127.7 ($2CH_{arom.}$), 127.5 ($CH_{arom.}$), 122.2 ($CH_{arom.}$), 122.1 ($CH_{arom.}$), 121.9 ($CH_{arom.}$), 121.4 (C_q), 120.9 ($CH_{arom.}$), 100.6 (C_q), 92.5 (C_q), 90.4 (C_q), 89.7 ($4'_{dU}$), 86.9 ($1'_{dU}$), 71.4 ($3'_{dU}$), 62.3 ($5'_{dU}$), 42.6 ($2'_{dU}$).

HR-MS (ESI): m/z calculated for $C_{31}H_{22}O_5N_2^+$ [M$^+$] = 502.1529; found = 502.15192.

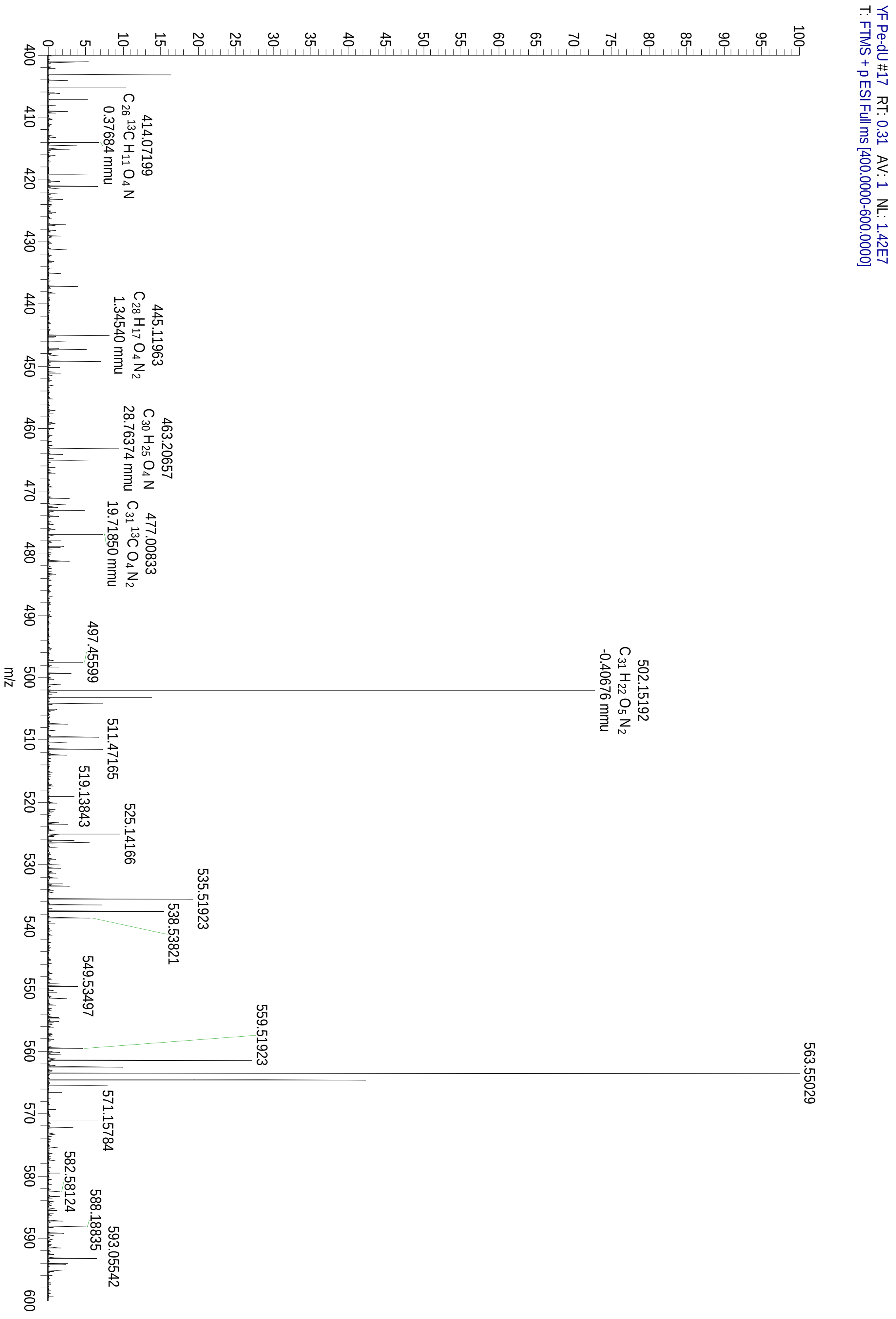

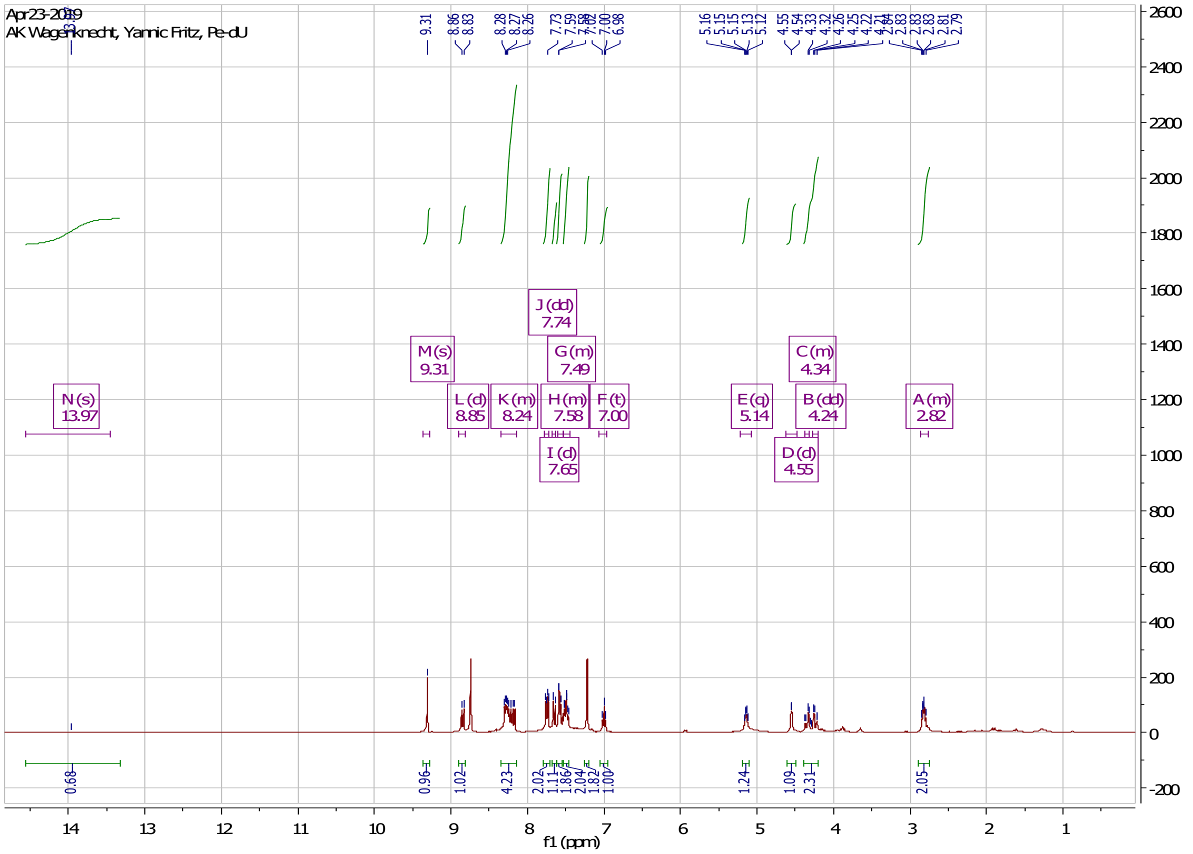
Apr23-2019
AK Wagenknecht, Yannic Fritz, Pe-dU
N (s) 13.97
M (s) 9.31
L (d) 8.85
K (m) 8.24
J (dd) 7.74
I (d) 7.65
H (m) 7.58
G (m) 7.49
F (t) 7.00
E (q) 5.14
D (d) 4.55
C (m) 4.34
B (dd) 4.24
A (m) 2.82
f1 (ppm)

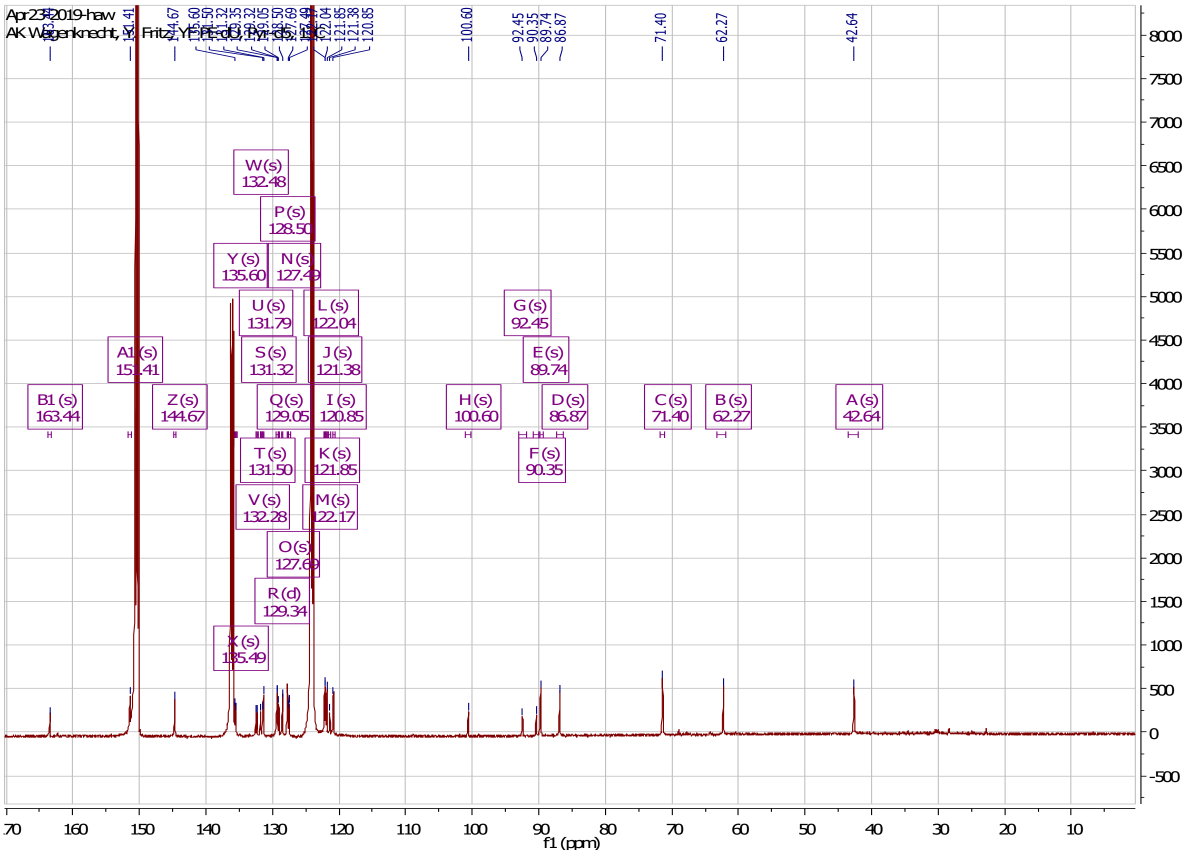
Apr23-2019-haw
B1 (s) 163.44
A1 (s) 151.41
Z (s) 144.67
Y (s) 135.60
X (s) 135.49
W (s) 132.48
V (s) 132.28
U (s) 131.79
T (s) 131.50
S (s) 131.32
R (d) 129.34
Q (s) 129.05
P (s) 128.50
O (s) 127.69
N (s) 127.49
M (s) 122.17
L (s) 122.04
K (s) 121.85
J (s) 121.38
I (s) 120.85
H (s) 100.60
G (s) 92.45
F (s) 90.35
E (s) 89.74
D (s) 86.87
C (s) 71.40
B (s) 62.27
A (s) 42.64
f1 (ppm)

2-Amino-8-iodo-2'-deoxyadenosine (8)

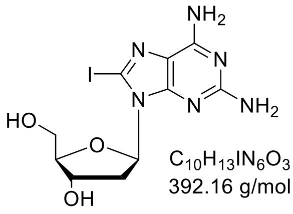

$C_{10}H_{13}IN_6O_3$
392.16 g/mol

8

2-amino-2'-deoxyadenosine (500 mg, 1.88 mmol, 1.00 eq.) was dissolved in DMF (15 mL) and cooled to 0 °C. N-iodo-succinimide (610 mg, 2.71 mmol, 1.44 eq.) and a few drops trifluoracetic acid were added.(Castanet et al., 2002) The mixture was stirred at room temperature overnight. The solvent was removed under reduced pressure and the crude product was purified by silica gel column chromatography (CH_2Cl_2:MeOH 15:1 – 5:1). The product was isolated as a white solid in a yield of 61% (487 mg, 1.24 mmol).

TLC R_f(CH_2Cl_2:MeOH 5:1) = 0.46

^{1}H NMR (500 MHz, DMSO-d_6): δ (ppm) = 7.17 (s, $2H_{amino}$), 6.11 (dd, *J* = 8.6, 6.2 Hz, 1H, $1'_{daA}$), 5.89 (s, $2H_{amino}$), 5.30 (s,$1H_{OH}$), 4.42 (q, *J* = 2.8 Hz, 1H, $3'_{daA}$), 3.88 (td, *J* = 4.1, 2.0 Hz, 1H, $4'_{daA}$), 3.67 (dd, *J* = 11.9, 4.3 Hz, 1H, $5'_{daA}$), 3.52 (dd, *J* = 11.9, 4.3 Hz, 1H, $5'_{daA}$), 3.17 (ddd, *J* = 13.0, 8.7, 5.9 Hz, 1H, $2'_{daA}$), 2.11 – 2.01 (m, 1H, $2'_{daA}$).

^{13}C NMR (126 MHz, DMSO-d_6): δ (ppm) = 158.39 (C_q), 154.56 (C_q), 151.53 (C_q), 116.96 (C_q), 96.89 (C_q), 88.33 ($4'_{daA}$), 88.00 ($1'_{daA}$), 71.57 ($3'_{daA}$), 62.40 ($5'_{daA}$), 36.97 ($2'_{daA}$).

HR-MS (ESI): m/z calculated for $C_{10}H_{14}IO_3N_6^+$ [M-H$^+$] = 393.0172; found = 393.01617.

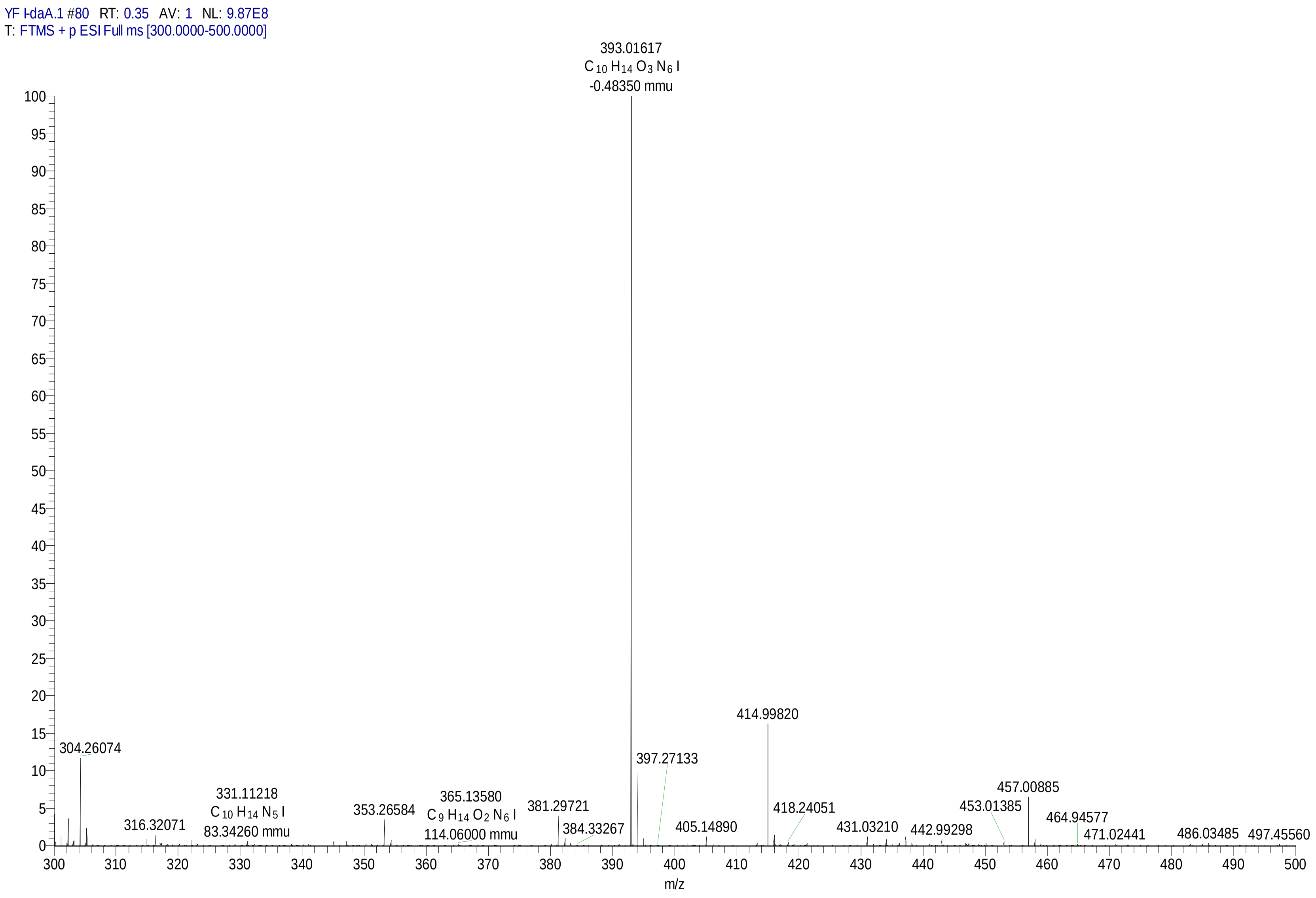
YF I-daA.1 #80 RT: 0.35 AV: 1 NL: 9.87E8
T: FTMS + p ESI Full ms [300.0000-500.0000]
393.01617
C 10 H 14 O 3 N 6 I
-0.48350 mmu
414.99820
304.26074
397.27133
331.11218
C 10 H 14 N 5 I
83.34260 mmu
353.26584
365.13580
C 9 H 14 O 2 N 6 I
114.06000 mmu
381.29721
384.33267
316.32071
405.14890
418.24051
431.03210
442.99298
453.01385
457.00885
464.94577
471.02441
486.03485
497.45560
m/z

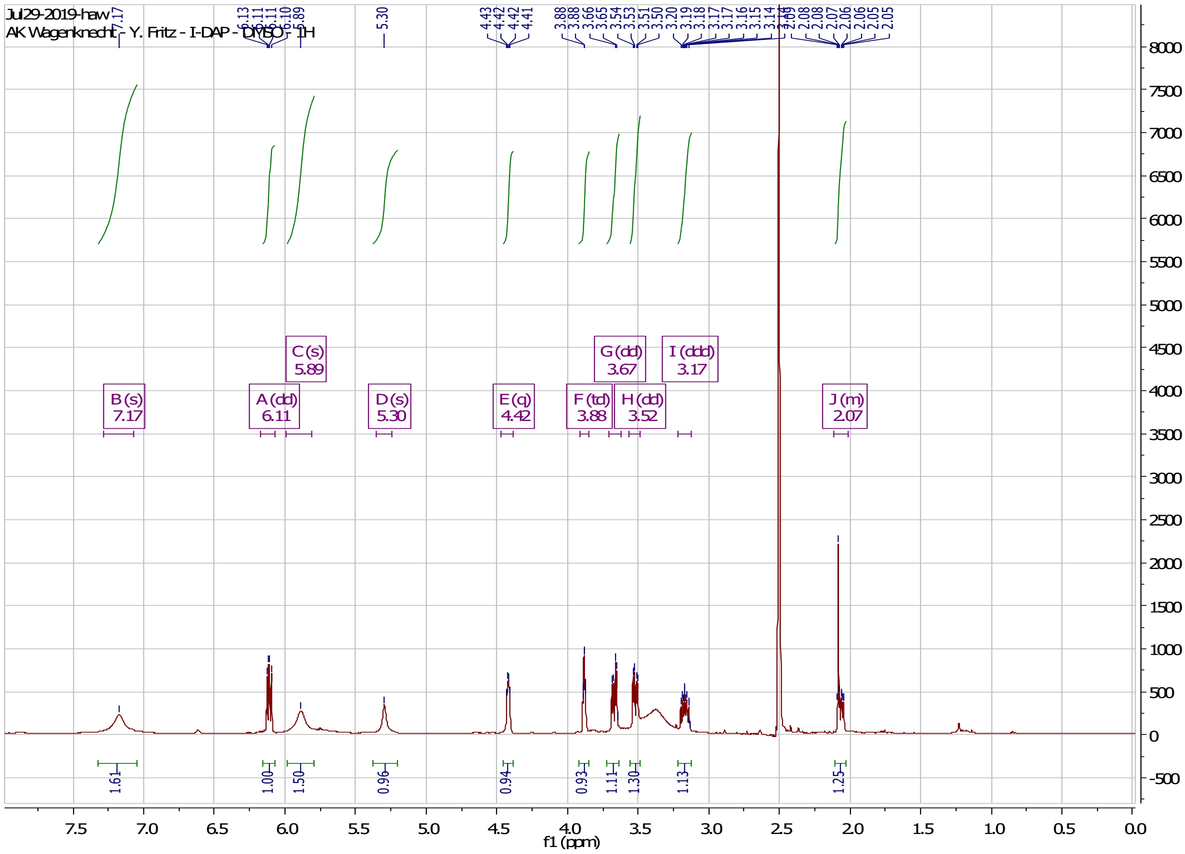
Jul29-2019-haw
AK Wagenknecht - Y. Fritz - I-DAP - DMSO - 1H
B (s) 7.17
A (dd) 6.11
C (s) 5.89
D (s) 5.30
E (q) 4.42
F (td) 3.88
G (dd) 3.67
H (dd) 3.52
I (ddd) 3.17
J (m) 2.07
f1 (ppm)

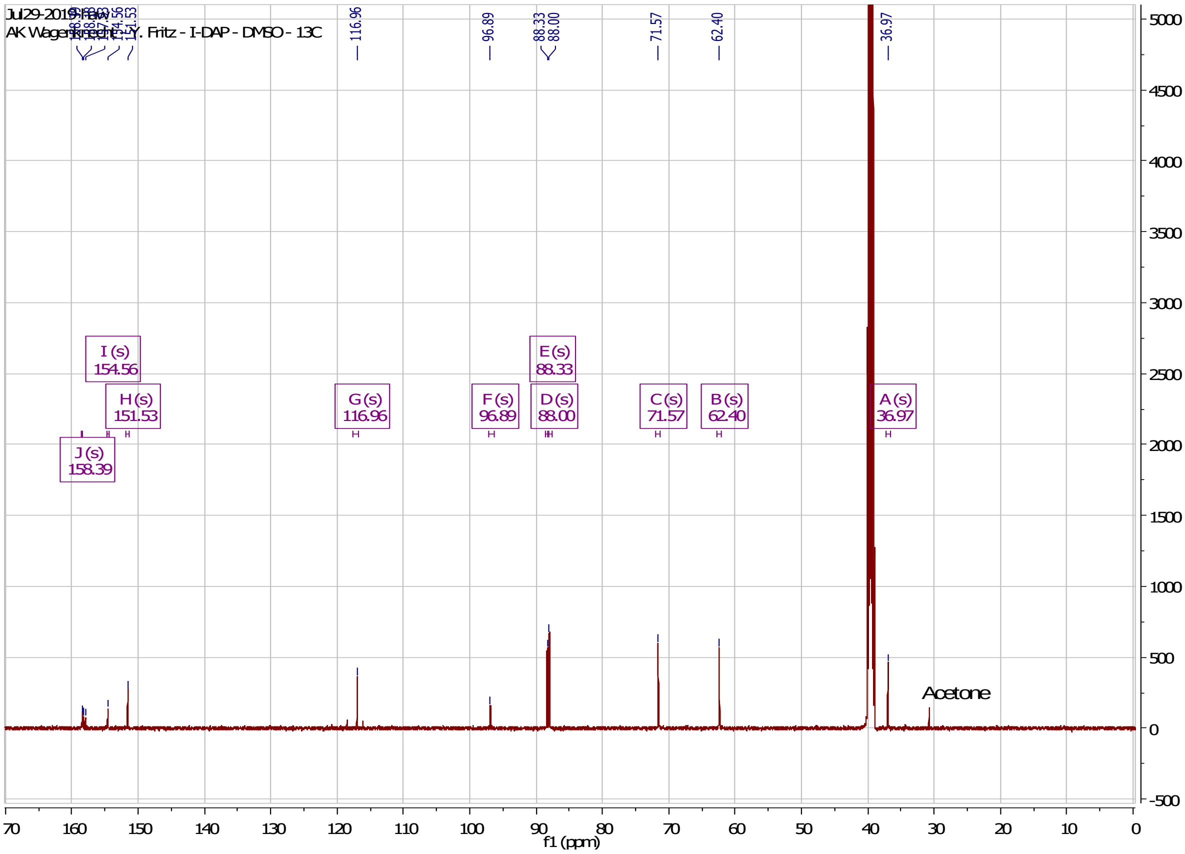
Jul29-2019-haw
AK Wagenknecht - Y. Fritz - I-DAP - DMSO - 13C
I (s) 154.56
H (s) 151.53
J (s) 158.39
G (s) 116.96
F (s) 96.89
E (s) 88.33
D (s) 88.00
C (s) 71.57
B (s) 62.40
A (s) 36.97
Acetone
f1 (ppm)

3',5'-Bis(*tert*-Butyldimethylsilyloxy)-2-amino-8-iodo-2'-deoxyadenosine (9)

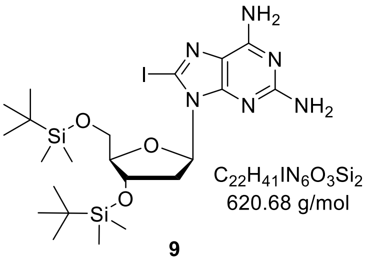

9

8 (1.14 g, 2.91 mmol, 1.00 eq.) and imidazole (792 mg, 11.6 mmol, 4.00 eq.) were dissolved in dry DMF (15 mL) and degassed. TBDMS-Cl (1.26 g, 8.36 mmol, 2.87 eq.) was added and the mixture stirred at room temperature overnight. The same volume water was added and extracted with hexane. The combined organic fractions were dried over Na_2SO_4 and the solvent removed under reduced pressure. The product was isolated as white solid with quantitative yield (1.80 g, 2.90 mmol).

TLC R_f(CH_2Cl_2:MeOH 10:1) = 0.58

^{1}H NMR (500 MHz, $CDCl_3$): δ (ppm) = 6.14 (t, *J* = 6.8 Hz, 1H, $1'_{daA}$), 5.64 (s, $2H_{amino}$), 4.84 – 4.73 (m, 1H, $3'_{daA}$), 4.67 (s, $2H_{amino}$), 3.92 (ddd, *J* = 7.0, 4.7, 3.3 Hz, 1H, $4'_{daA}$), 3.87 (dd, *J* = 10.6, 7.0 Hz, 1H, $5'_{daA}$), 3.68 (dd, *J* = 10.6, 4.7 Hz, 1H, $5'_{daA}$), 3.62 (dt, *J* = 13.0, 6.4 Hz, 1H, $2'_{daA}$), 2.13 (ddd, *J* = 13.0, 6.9, 3.9 Hz, 1H, $2'_{daA}$), 0.93 (s, $9H_{TBDMS}$), 0.85 (s, $9H_{TBDMS}$), 0.13 (s, $6H_{TBDMS}$), -0.01 (d, *J* = 16.8 Hz, $6H_{TBDMS}$).

^{13}C NMR (126 MHz, $CDCl_3$): δ (ppm) = 159.07 (C_q), 154.70 (C_q), 152.65 (C_q), 118.60 (C_q), 95.83 (C_q), 87.95 ($1'_{daA}$), 87.60 ($4'_{daA}$), 72.72 ($3'_{daA}$), 62.95 ($5'_{daA}$), 36.34 ($2'_{daA}$), 26.00 ($6C_{3,TBDMS}$), 18.49 ($C_{q,TBDMS}$), 18.17 ($C_{q,TBDMS}$), -4.49 ($2CH_{3,TBDMS}$), -5.24 ($2CH_{3,TBDMS}$).

HR-MS (ESI): m/z calculated for $C_{22}H_{42}IO_3N_6Si_2^+$ [M-H$^+$] = 621.1902; found = 621.18896.

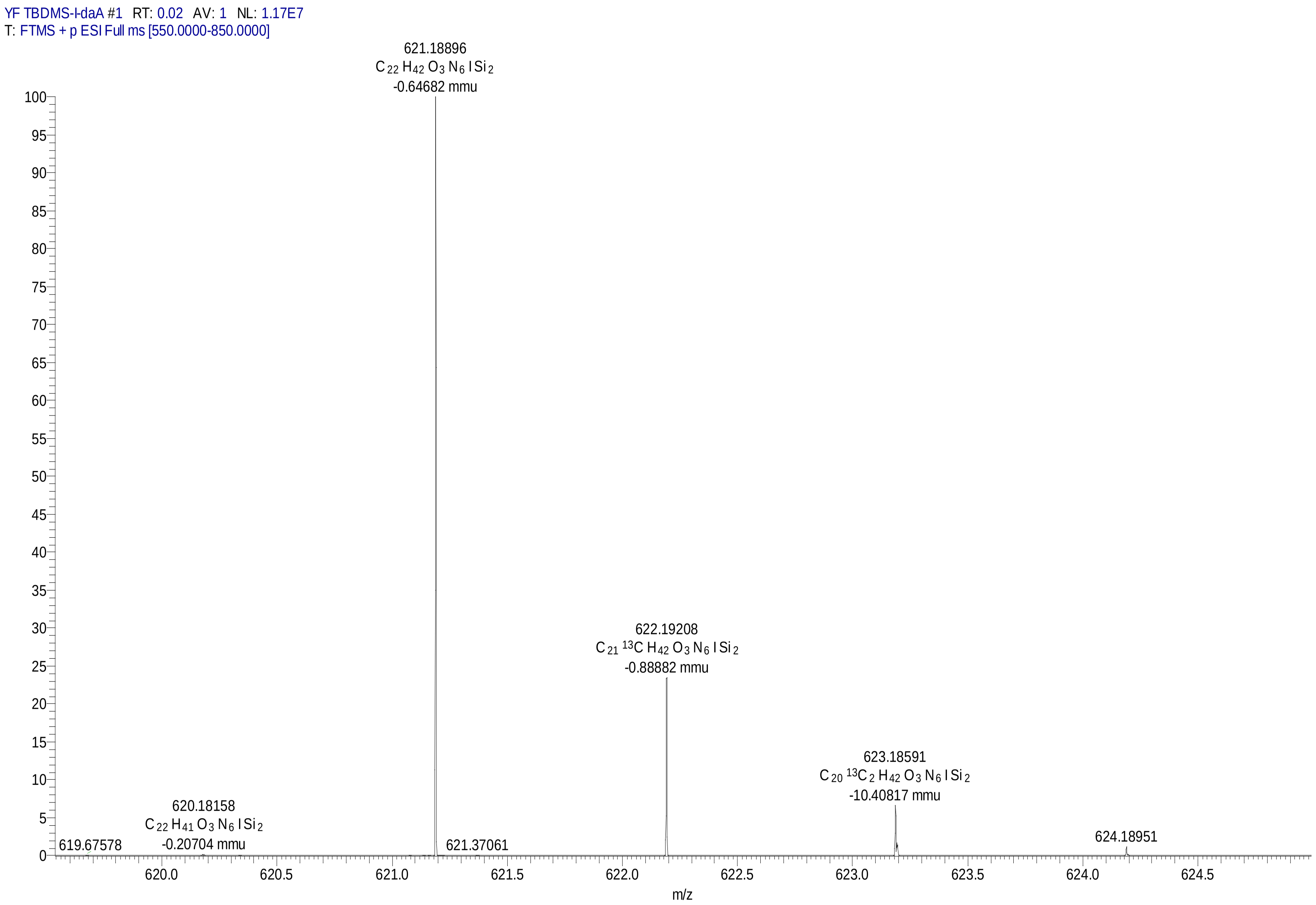

YF TBDMS-I-daA #1 RT: 0.02 AV: 1 NL: 1.17E7
T: FTMS + p ESI Full ms [550.0000-850.0000]
621.18896
C 22 H42 O3 N6 I Si 2
-0.64682 mmu
622.19208
C 21 13C H42 O3 N6 I Si 2
-0.88882 mmu
623.18591
C 20 13C 2 H42 O3 N6 I Si 2
-10.40817 mmu
620.18158
C 22 H41 O3 N6 I Si 2
-0.20704 mmu
619.67578
621.37061
624.18951
m/z

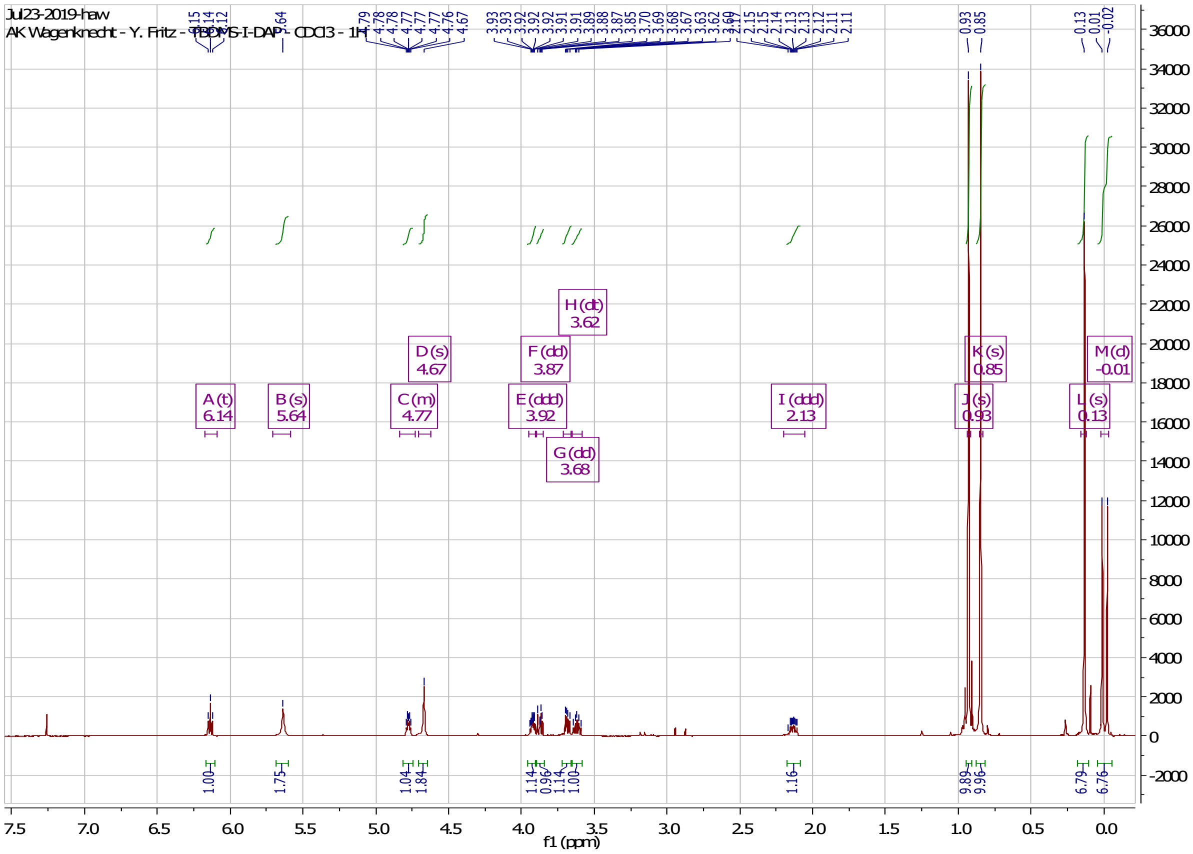

Jul23-2019-haw
AK Wagenknecht - Y. Fritz - TBDMS-I-DAP - CDCl3 - 1H
A (t) 6.14
B (s) 5.64
C (m) 4.77
D (s) 4.67
E (ddd) 3.92
F (dd) 3.87
G (dd) 3.68
H (dt) 3.62
I (ddd) 2.13
J (s) 0.93
K (s) 0.85
L (s) 0.13
M (d) -0.01
f1 (ppm)

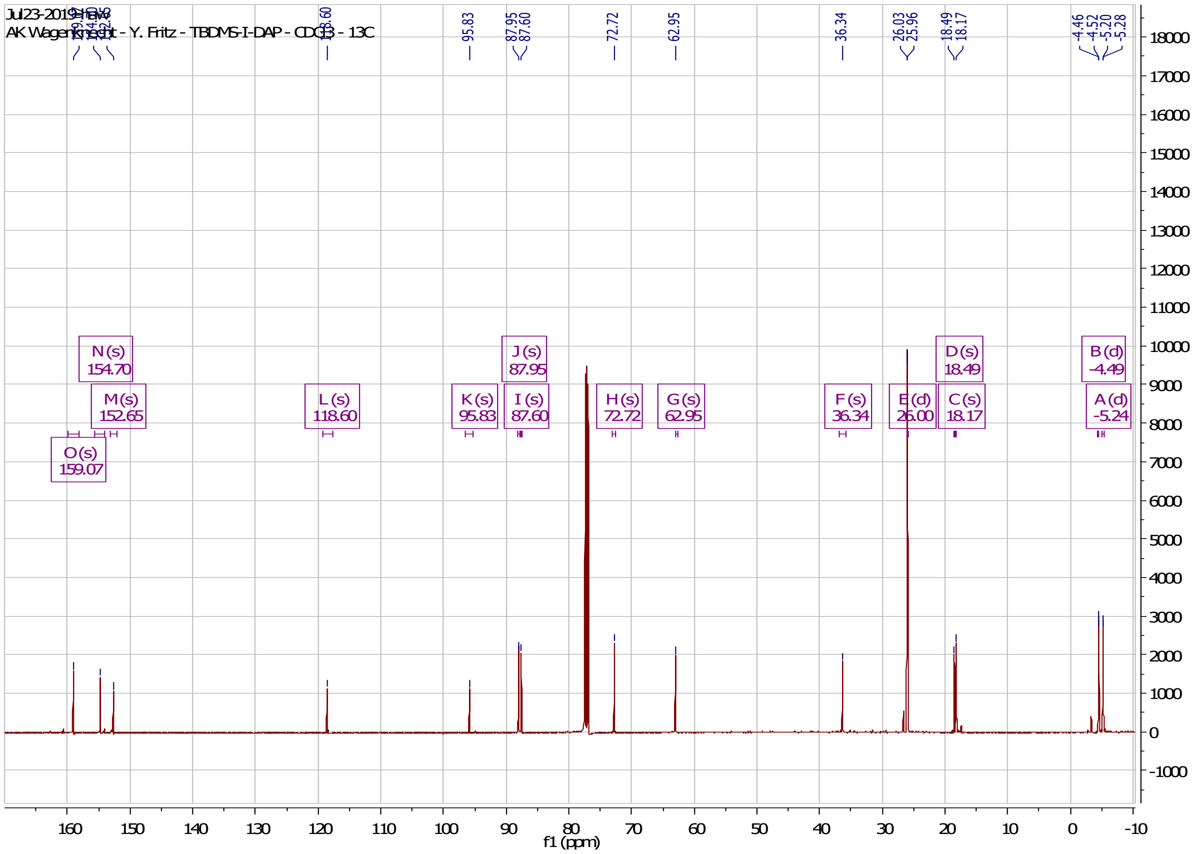

Jul23-2019-haw
AK Wagenknecht - Y. Fritz - TBDMS-I-DAP - CDCl3 - 13C
N (s) 154.70
M (s) 152.65
O (s) 159.07
L (s) 118.60
K (s) 95.83
J (s) 87.95
I (s) 87.60
H (s) 72.72
G (s) 62.95
F (s) 36.34
E (d) 26.00
D (s) 18.49
C (s) 18.17
B (d) -4.49
A (d) -5.24
f1 (ppm)

2-Amino-8-(perylene-3-ylethynyl)-2‘-deoxyadenosine (Pe-Et-daA)

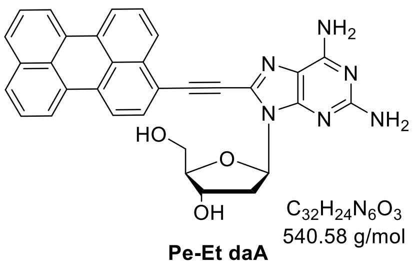

Pe-Et daA

5 (72 mg, 261 µmol, 1.00 eq.) and **9** (162 mg, 261 µmol, 1.00 eq.) were suspended in NEt_3 (4 mL) and dioxane (4 mL) in a 10 mL vial. The suspension was degassed via freeze-pump-thaw and tetrakis(triphenylphosphine)palladium (10 mg, 8.65 µmol, 0.03 eq.) was added. The sealed vial was heated to 90 °C and the solution was stirred overnight. The solvent was removed under reduced pressure and the crude product was purified by silica gel column chromatography (CH_2Cl_2:acetone 1:0 – 1:1, R_f(EtOAc) = 0.68). The TBDMS-protected product was dissolved in THF (3 mL) and NEt_3*3HF (179 µL, 177 mg, 1.10 mmol, 4.21 eq.) were added. The mixture was stirred in a sealed vial at room temperature overnight. The reaction was stopped by adding silica gel and the solvent was removed under reduced pressure. The crude product was purified by silica gel column chromatography (CH_2Cl_2:MeOH 50:1 – 10:1). The product was isolated as yellow solid with a yield of 30% (43 mg, 79.5 µmol).

TLC R_f(CH_2Cl_2:MeOH 10:1) = 0.37

^{1}H NMR (500 MHz, Pyr-d_5): δ (ppm) = 8.44 (d, *J* = 8.2 Hz, $1H_{arom.}$), 8.36 (s, $2H_{amino}$), 8.28 (dd, *J* = 7.7, 2.6 Hz, $2H_{arom.}$), 8.23 (d, *J* = 7.5 Hz, $1H_{arom.}$), 8.11 (d, *J* = 7.9 Hz, $1H_{arom.}$), 7.77 (t, *J* = 7.8 Hz, $2H_{arom.}$), 7.66 (d, *J* = 7.8 Hz, $1H_{arom.}$), 7.64 – 7.59 (t, *J* = 7.9 Hz, $1H_{arom.}$), 7.51 (td, *J* = 7.8, 2.8 Hz, $2H_{arom.}$), 7.39 (dd, *J* = 9.1, 5.9 Hz, 1H, $1'_{daA}$), 6.84 (s, $2H_{amino}$), 5.26 (d, *J* = 5.1 Hz, 1H, $3'_{daA}$), 4.75 (t, *J* = 2.3 Hz, 1H, $4'_{daA}$), 4.35 (dd, *J* = 12.3, 2.6 Hz, 1H, $5'_{daA}$), 4.22 – 4.10 (m, 1H, $5'_{daA}$), 3.72 (ddd, *J* = 13.2, 9.4, 5.6 Hz, 1H, $2'_{daA}$), 2.73 (dd, *J* = 13.0, 5.8 Hz, 1H, $2'_{daA}$).

^{13}C NMR (126 MHz, Pyr-d_5): δ (ppm) = 162.4 ($CH_{arom.}$), 158.7 ($CH_{arom.}$), 152.2 (C_q), 135.5 (C_q), 135.4 (C_q), 133.7 (C_q), 132.6 ($CH_{arom.}$), 132.5 (C_q), 131.8 (C_q), 131.5 (C_q), 131.1 (C_q), 129.7 (C_q), 129.2 (2C_q), 129.1 (C_q), 128.8 ($CH_{arom.}$), 127.7 (2$CH_{arom.}$), 126.5 ($CH_{arom.}$), 122.6 ($CH_{arom.}$), 122.1 (2$CH_{arom.}$), 120.7 ($CH_{arom.}$), 118.8 (C_q), 117.1 (C_q), 93.7 (C_q), 91.1 ($4'_{daA}$), 88.1 ($1'_{daA}$), 86.6 (C_q), 73.8 ($3'_{daA}$), 64.7 ($5'_{daA}$), 40.4 ($2'_{daA}$).

HR-MS (ESI): m/z calculated for $C_{32}H_{25}O_3N_6^+$ [M-H$^+$] = 541.1988; found = 541.19575.

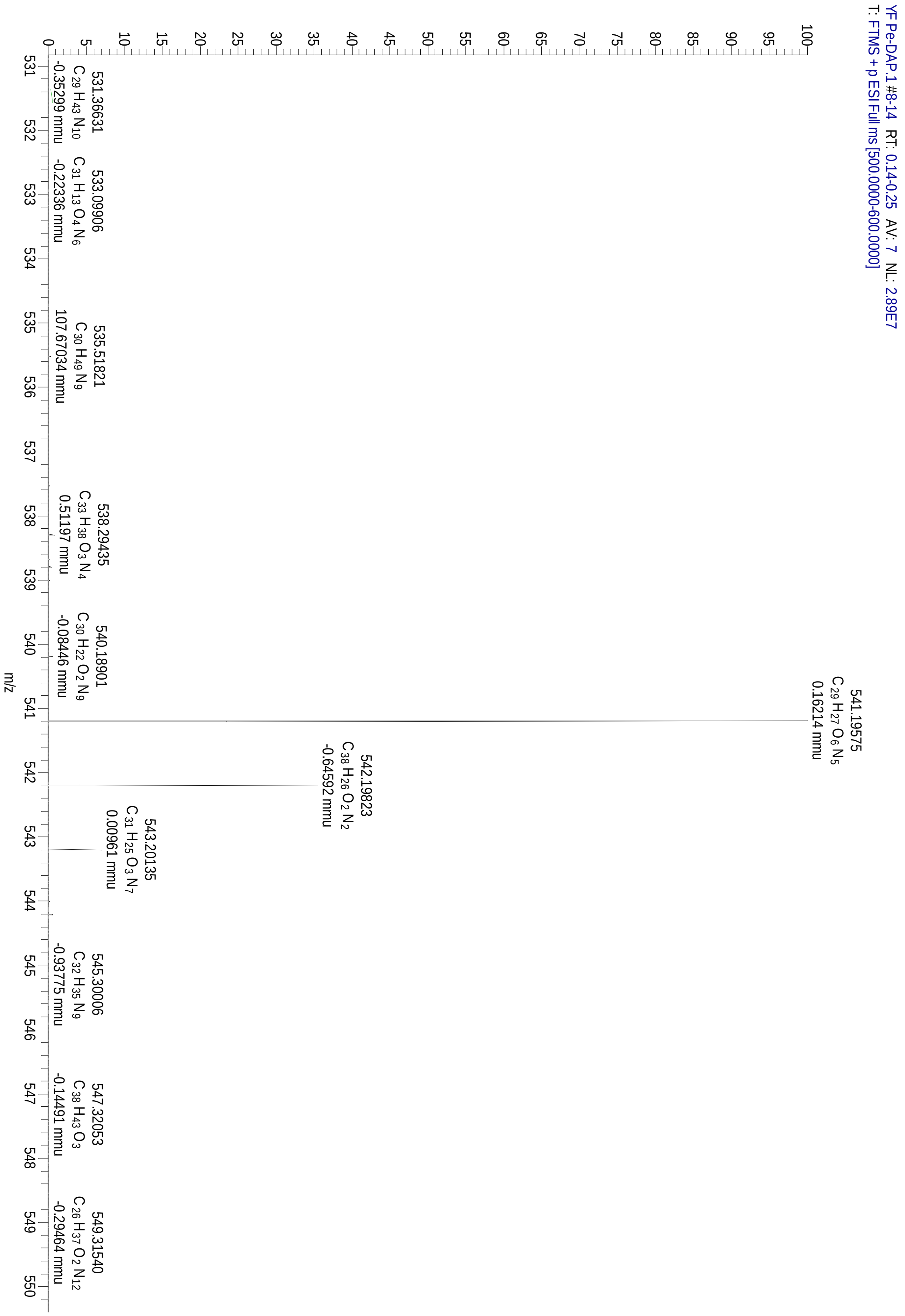

YF Pe-DAP.1 #8-14 RT: 0.14-0.25 AV: 7 NL: 2.89E7
T: FTMS + p ESI Full ms [500.0000-600.0000]
541.19575
C29 H27 O6 N5
0.16214 mmu
542.19823
C38 H26 O2 N2
-0.64592 mmu
543.20135
C31 H25 O3 N7
0.00961 mmu
531.36631
C29 H43 N10
-0.35299 mmu
533.09906
C31 H13 O4 N6
-0.22336 mmu
535.51821
C30 H49 N9
107.67034 mmu
538.29435
C33 H38 O3 N4
0.51197 mmu
540.18901
C30 H22 O2 N9
-0.08446 mmu
545.30006
C32 H35 N9
-0.93775 mmu
547.32053
C38 H43 O3
-0.14491 mmu
549.31540
C26 H37 O2 N12
-0.29464 mmu
m/z

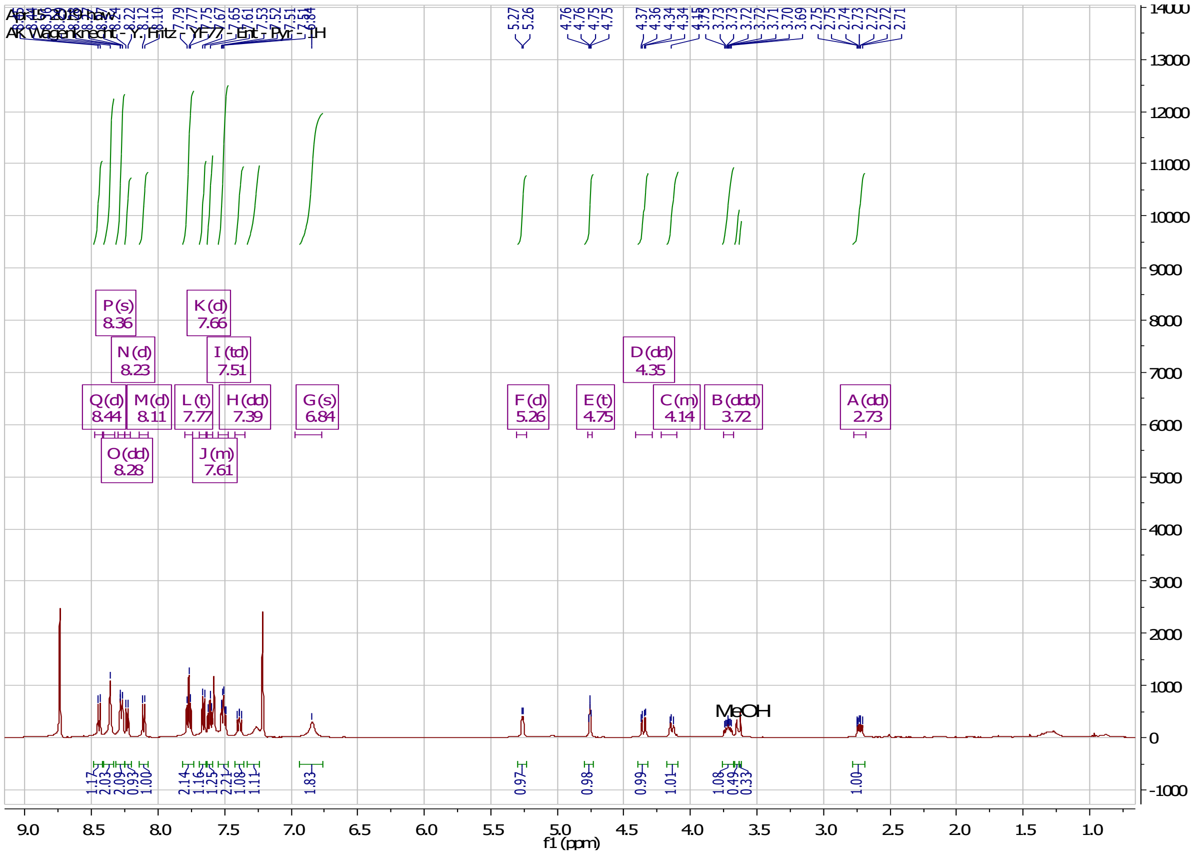

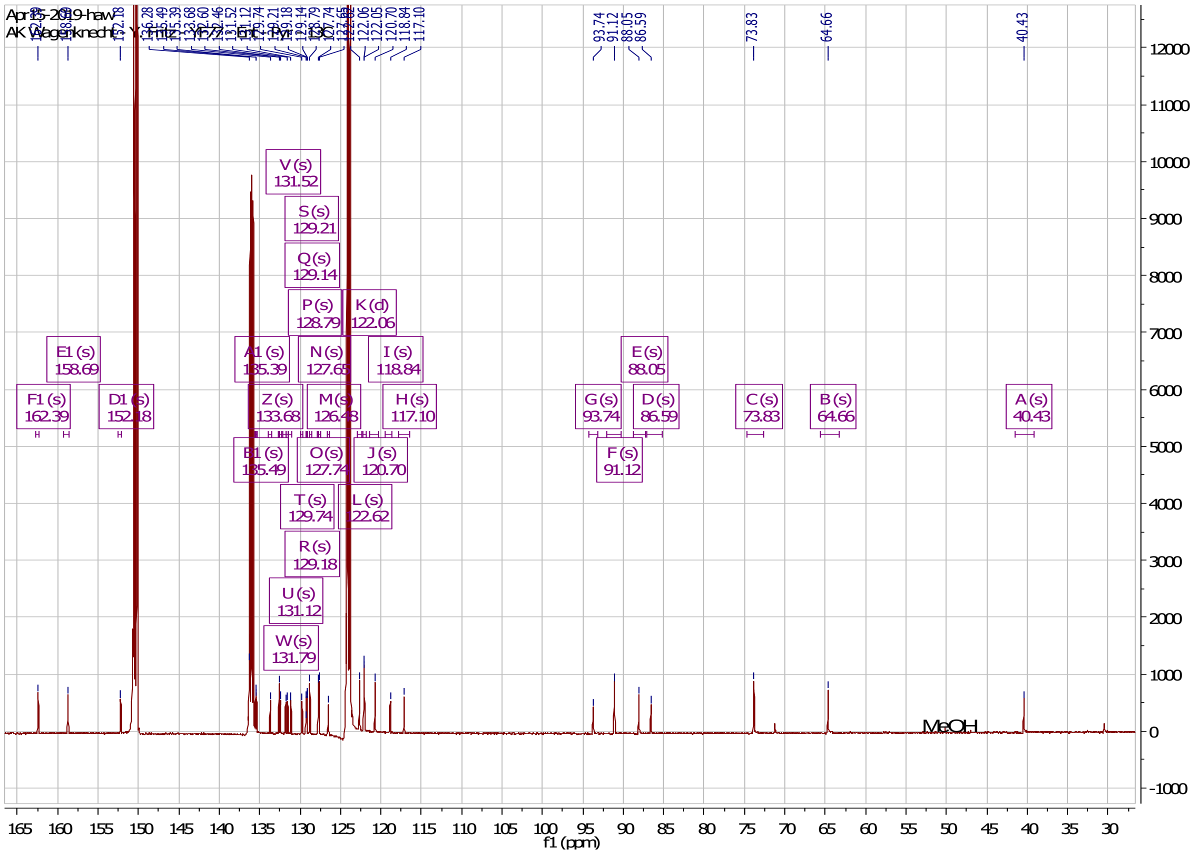

3-(*p*-bromophenyl)-perylene (10)

$C_{26}H_{15}Br$
407.31 g/mol

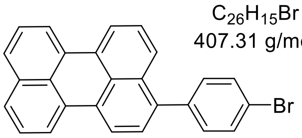

10

3 (730 mg, 1.93 mmol, 1.00 eq.), *p*-iodobromobenzene (734 mg, 2.59 mmol, 1.34 eq.) and NaO^tBu (627 mg, 6.52 mmol, 3.38 eq.) were suspended in THF (10 mL) and water (10 mL). The suspension was degassed via freeze-pump-thaw and tetrakis(triphenylphosphine)palladium (100 mg, 8.65 mmol, 0.04 eq.) was added. The mixture was stirred in a sealed vial at 80 °C overnight. The solvent was removed under reduced pressure and the crude solid dissolved in CH_2Cl_2. The organic layer was washed with $NaHCO_3$ solution, water and brine. After drying over Na_2SO_4, the solvent was removed under reduced pressure. The crude product was purified by silica gel column chromatography (hexane) and the product was isolated as yellow solid with a yield of 91% (716 mg, 1.76 mmol).

TLC R_f(hexane:$CHCl_3$ 3:1) = 0.63

^{1}H NMR (500 MHz, $CDCl_3$): δ (ppm) = 8.27 – 8.16 (m, 4H), 7.73 – 7.68 (m, 3H), 7.66 – 7.62 (m, 2H), 7.52 – 7.47 (m, 2H), 7.44 (dd, *J* = 8.4, 7.5 Hz, 1H), 7.41 – 7.38 (m, 3H).

^{13}C NMR (126 MHz, $CDCl_3$): δ (ppm) = 139.81 (C_q), 138.69 (C_q), 134.83 (C_q), 132.88 (C_q), 131.73 (4$CH_{arom.}$), 131.67 (C_q), 131.42 (C_q), 131.21 (2C_q), 129.23 (C_q), 128.76 (C_q), 128.09 (2$CH_{arom.}$), 127.81 ($CH_{arom.}$), 126.88 ($CH_{arom.}$), 126.80 (2$CH_{arom.}$), 125.80 ($CH_{arom.}$), 121.69 (C_q), 120.60 (2$CH_{arom.}$), 120.41 ($CH_{arom.}$), 119.99 ($CH_{arom.}$).

HR-MS (ESI): m/z calculated for $C_{26}H_{15}Br^+$ [M^+] = 406.0357; found = 406.03494.

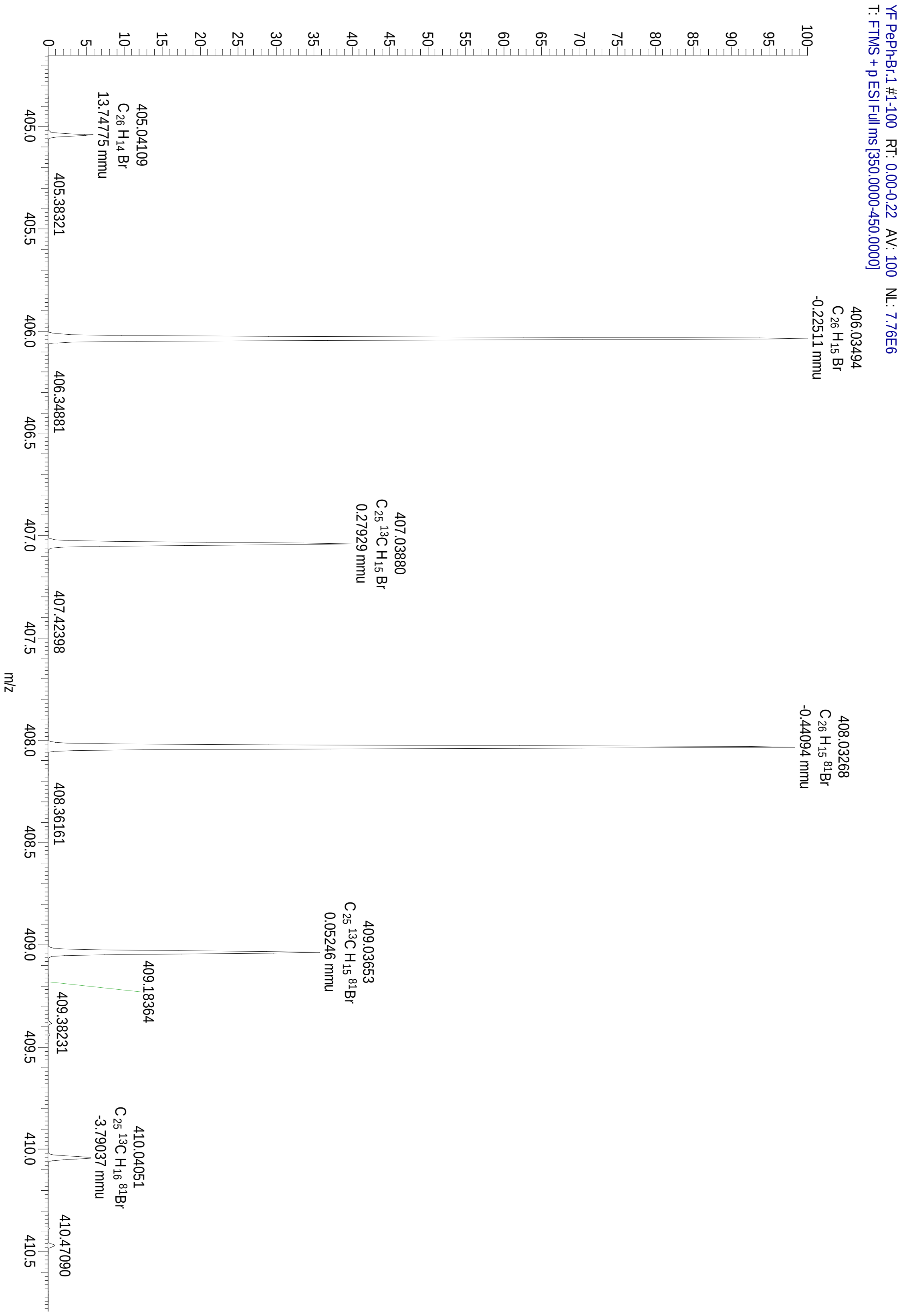
YF PePh-Br.1 #1-100 RT: 0.00-0.22 AV: 100 NL: 7.76E6
T: FTMS + p ESI Full ms [350.0000-450.0000]
405.04109
C26 H14 Br
13.74775 mmu
405.38321
406.03494
C26 H15 Br
-0.22511 mmu
406.34881
407.03880
C25 13C H15 Br
0.27929 mmu
407.42398
408.03268
C26 H15 81Br
-0.44094 mmu
408.36161
409.03653
C25 13C H15 81Br
0.05246 mmu
409.18364
409.38231
410.04051
C25 13C H16 81Br
-3.79037 mmu
410.47090
m/z

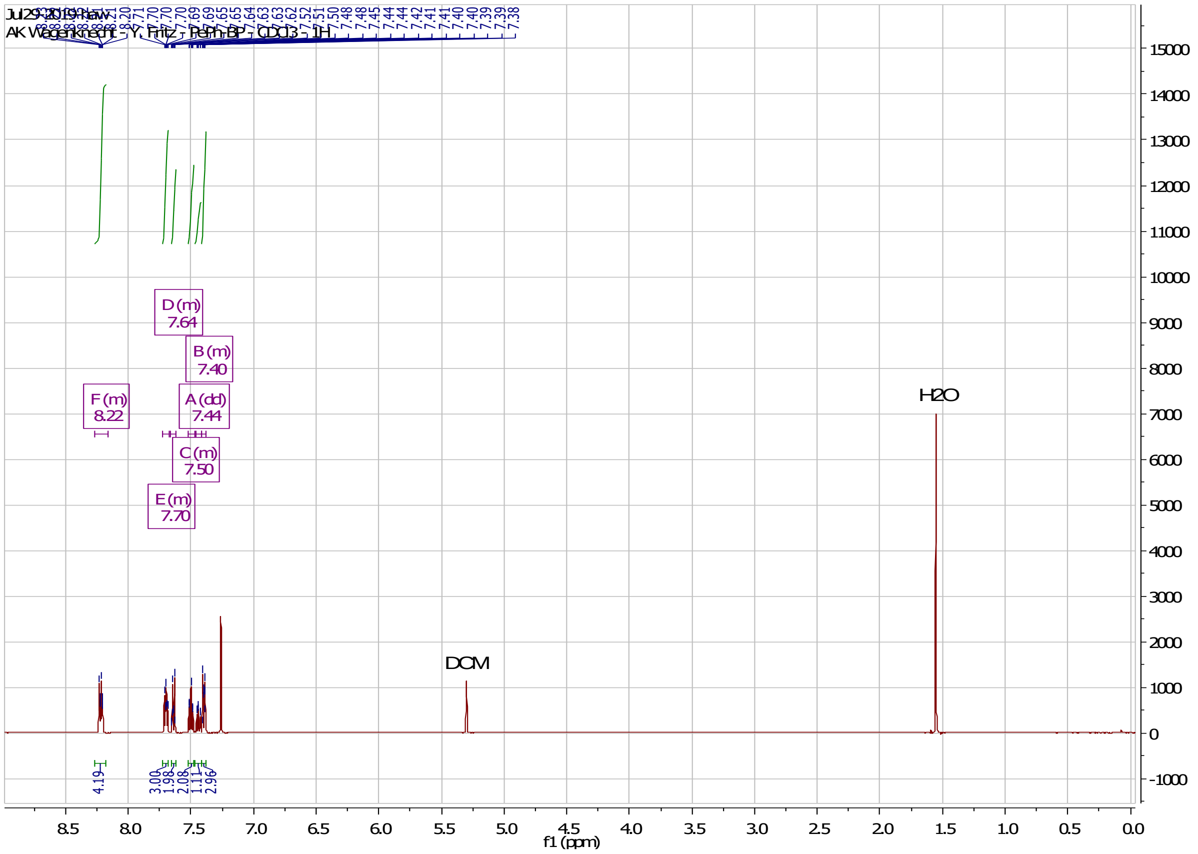
Jul29-2019-Raw
AK Wagenknecht - Y. Fritz - Perm-BP - CDCl3 - 1H
F (m) 8.22
D (m) 7.64
B (m) 7.40
A (dd) 7.44
C (m) 7.50
E (m) 7.70
DCM
H2O
4.19
3.00
1.98
2.08
1.11
2.96
f1 (ppm)

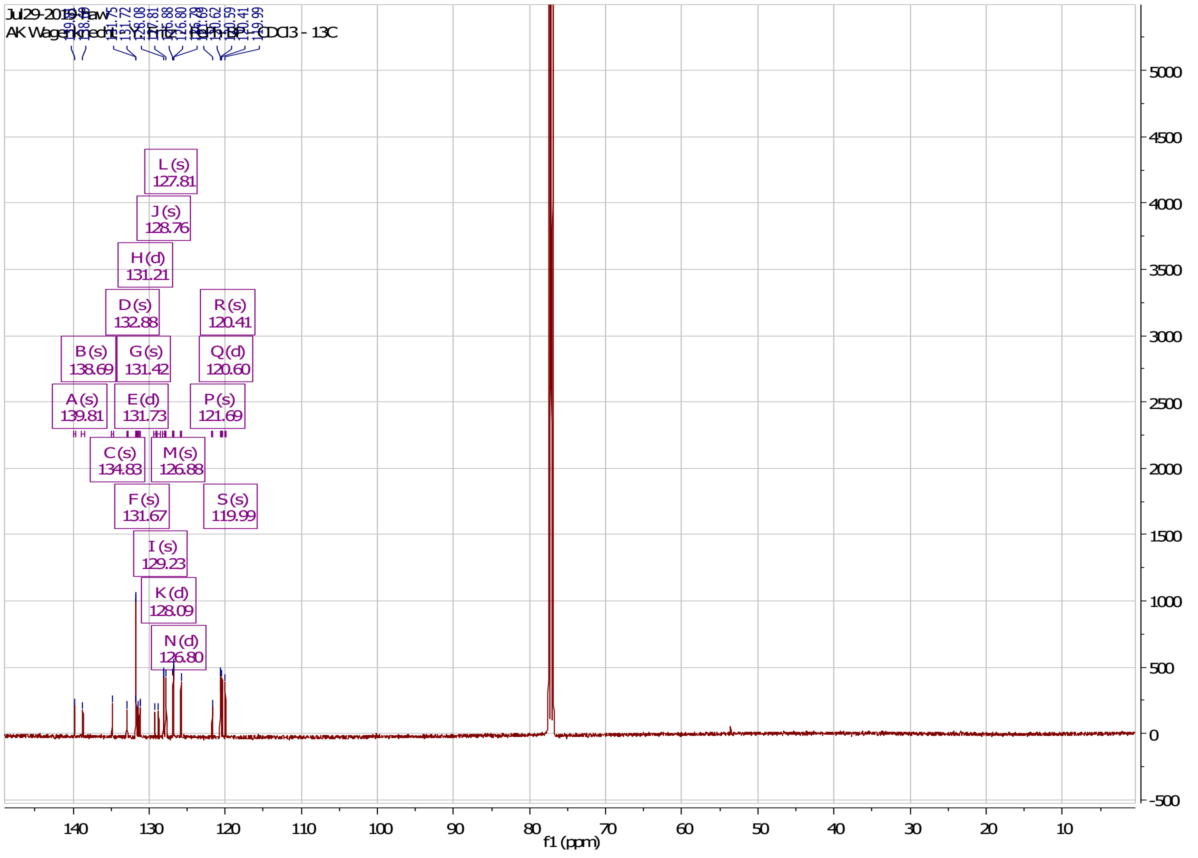
Jul29-2019-Raw
AK Wagenknecht - Y. Fritz - Perm-BP - CDCl3 - 13C
L (s) 127.81
J (s) 128.76
H (d) 131.21
D (s) 132.88
R (s) 120.41
B (s) 138.69
G (s) 131.42
Q (d) 120.60
A (s) 139.81
E (d) 131.73
P (s) 121.69
C (s) 134.83
M (s) 126.88
F (s) 131.67
S (s) 119.99
I (s) 129.23
K (d) 128.09
N (d) 126.80
f1 (ppm)

3-(*p*-Pinacolatoboronylphenyl)-perylene (11)

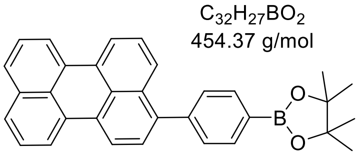

11

10 (504 mg, 1.24 mmol, 1.00 eq.), bis(pinacolato)diboron (871 mg, 3.43 mmol, 2.77 eq.), KOAc (363 mg, 3.69 mmol, 2.99 eq.) and [bis(diphenylphosphino)ferrocene]dichloropalladium (62 mg, 84.7 µmol, 0.07 eq.) were dissolved in dry dioxane (30 mL) and stirred at 115 °C for 17 h. The solvent was removed under reduced pressure and the crude solid dissolved in CH_2Cl_2. The organic layer was washed with $NaHCO_3$ solution and brine. After drying over Na_2SO_4, the solvent was removed under reduced pressure and the crude product was purified by silica gel column chromatography (hexane:CH_2Cl_2 1:1). The product was isolated as yellow solid with a yield of 55% (311 mg, 684 µmol).

TLC R_f(hexane:$CHCl_3$ 3:1) = 0.18

^{1}H NMR (500 MHz, $CDCl_3$) δ (ppm) = 8.30 – 8.14 (m, 4H), 8.04 – 7.91 (m, 2H), 7.75 (dd, *J* = 8.4, 0.9 Hz, 1H), 7.69 (dd, *J* = 8.3, 0.9 Hz, 2H), 7.57 – 7.53 (m, 2H), 7.49 (td, *J* = 7.9, 1.7 Hz, 2H), 7.45 – 7.39 (m, 2H), 1.40 (s, 12H).

^{13}C NMR (126 MHz, $CDCl_3$): δ (ppm) = 143.83 (C_q), 140.00 (C_q), 134.97 (2$CH_{arom.}$), 134.84 ($C_{q.}$), 132.96 (C_q), 131.55 (2C_q), 131.38 (C_q), 130.93 (C_q), 129.51 (2$CH_{arom.}$), 129.20 (C_q), 128.80 (C_q), 127.95 (2$CH_{arom.}$), 127.82 ($CH_{arom.}$), 126.77 (2$CH_{arom.}$), 126.70 ($CH_{arom.}$), 126.20 ($CH_{arom.}$), 120.51 (2$CH_{arom.}$), 120.31 ($CH_{arom.}$), 120.05 ($CH_{arom.}$), 84.05 (3C_q), 25.07 (4CH_3).

HR-MS (ESI): m/z calculated for $C_{32}H_{27}BO_5^+$ [M^+] = 454.2104; found = 454.20978.

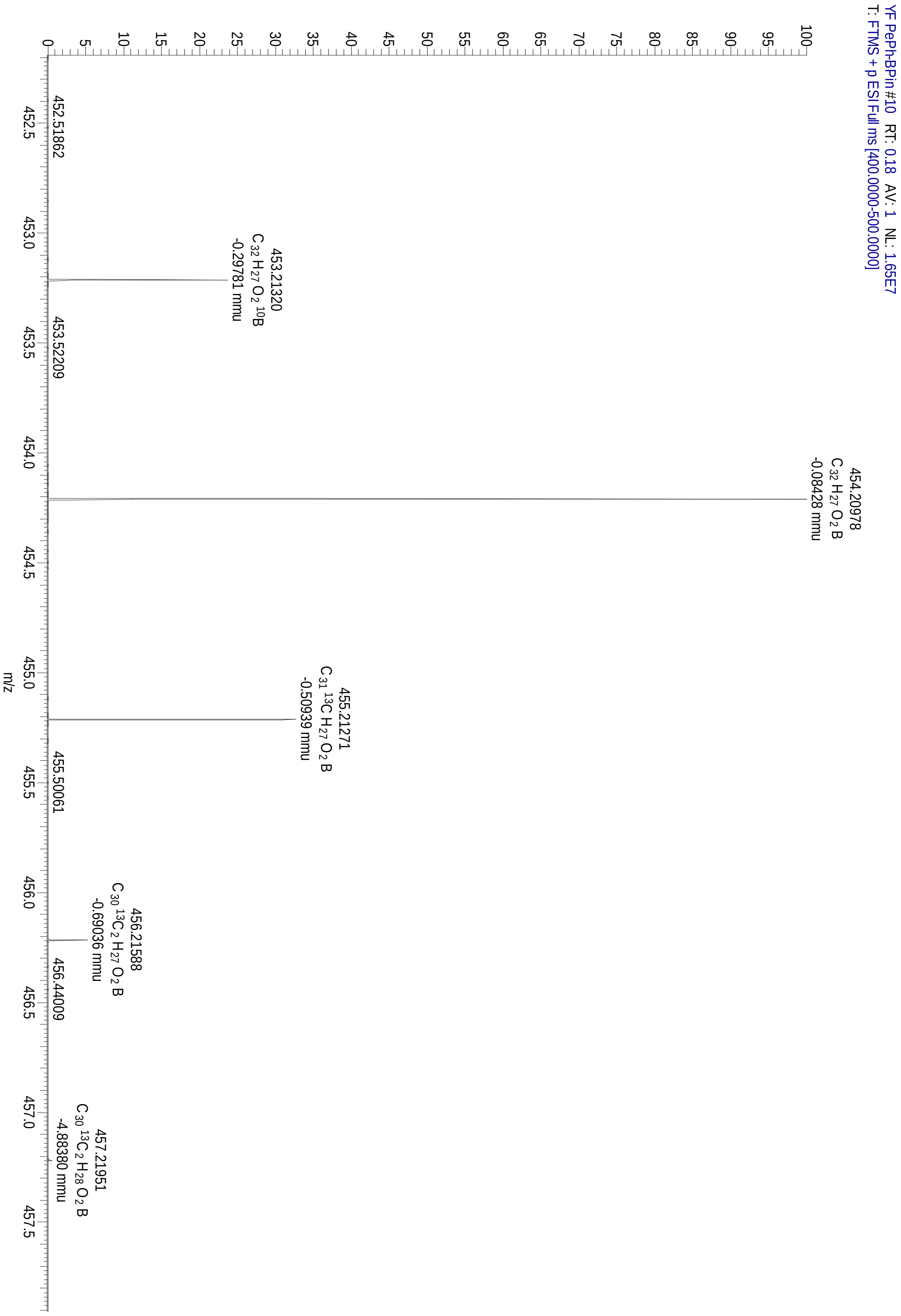
YF PePh-BPin #10 RT: 0.18 AV: 1 NL: 1.65E7
T: FTMS + p ESI Full ms [400.0000-500.0000]
452.51862
453.21320
C32 H27 O2 10B
-0.29781 mmu
453.52209
454.20978
C32 H27 O2 B
-0.08428 mmu
455.21271
C31 13C H27 O2 B
-0.50939 mmu
455.50061
456.21588
C30 13C2 H27 O2 B
-0.69036 mmu
456.44009
457.21951
C30 13C2 H28 O2 B
-4.88380 mmu
m/z

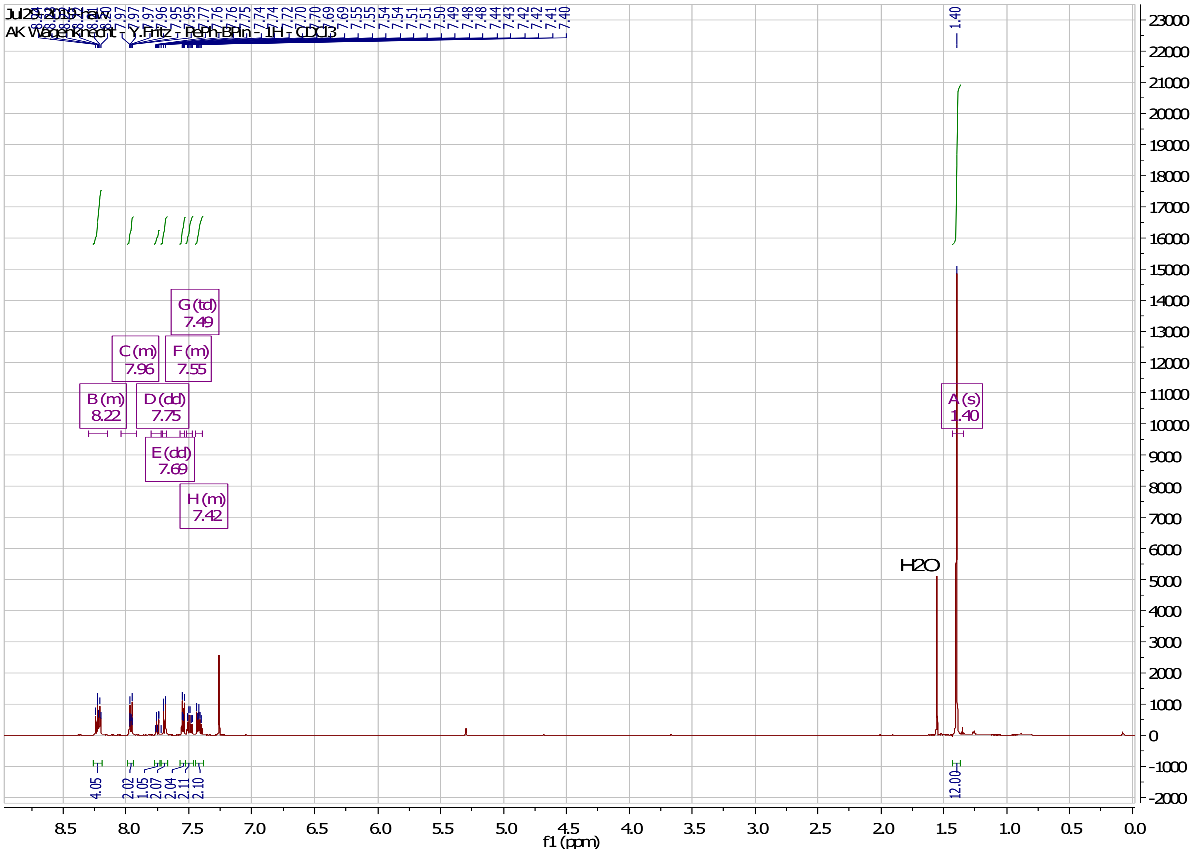
H2O
G (td) 7.49
C (m) 7.96
F (m) 7.55
B (m) 8.22
D (dd) 7.75
E (dd) 7.69
H (m) 7.42
A (s) 1.40
4.05
2.02
1.05
2.07
2.04
2.11
2.10
12.00
f1 (ppm)

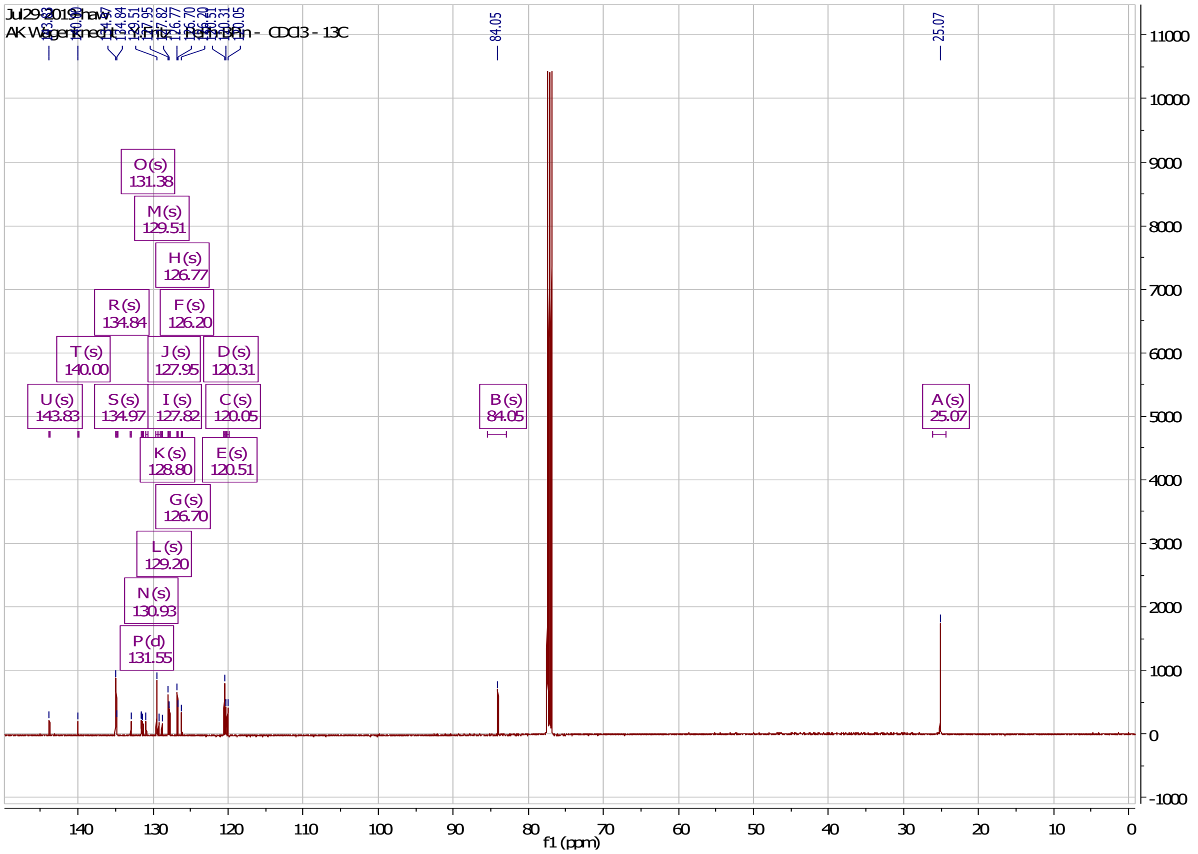
84.05
25.07
O (s) 131.38
M (s) 129.51
H (s) 126.77
R (s) 134.84
F (s) 126.20
T (s) 140.00
J (s) 127.95
D (s) 120.31
U (s) 143.83
S (s) 134.97
I (s) 127.82
C (s) 120.05
B (s) 84.05
A (s) 25.07
K (s) 128.80
E (s) 120.51
G (s) 126.70
L (s) 129.20
N (s) 130.93
P (d) 131.55
f1 (ppm)

5-(Perylene-3-yl-*p*-phenyl)-2‘-deoxyuridine (Pe-Ph-dU)

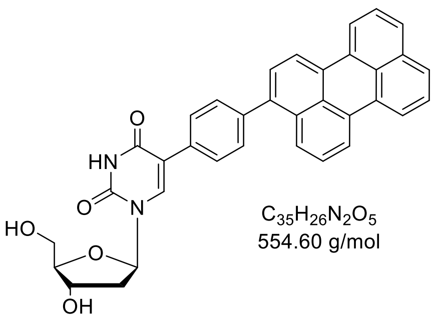

Pe-Ph-dU

11 (111 mg, 245 µmol, 1.00 eq.), **6** (110 mg, 311 µmol, 1.26 eq.) and NaO^{t}Bu (94 mg, 978 µmol, 3.99 eq.) were suspended in water (1 mL) and THF (15 mL) in a 20 mL vial. The suspension was degassed via freeze-pump-thaw and tetrakis(triphenylphosphine)palladium (10 mg, 8.65 µmol, 0.04 eq.) was added. The sealed vial was heated to 100 °C and the mixture was stirred overnight. The solvent was removed under reduced pressure and the crude solid dissolved in CH_2Cl_2. The organic layer was washed with $NaHCO_3$ solution and brine. After drying over Na_2SO_4, the solvent was removed under reduced pressure and the crude product was purified by silica gel column chromatography (CH_2Cl_2:MeOH 50:1 – 20:1). The product was isolated as yellow solid with a yield of 38% (52 mg, 95.2 µmol).

TLC R_f(CH_2Cl_2:MeOH 10:1) = 0.45

^{1}H NMR (500 MHz, Pyr-d_5): δ (ppm) = 13.20 (s, $1H_{imido}$), 9.13 (s, 1H, 6_{dU}), 8.37 (m, $4H_{arom.}$), 8.26 – 8.17 (m, $2H_{arom.}$), 7.85 (d, *J* = 8.4 Hz, $1H_{arom.}$), 7.78 (d, *J* = 8.1 Hz, $2H_{arom.}$), 7.57 – 7.50 (m, $4H_{arom.}$), 7.44 (m, $2H_{arom.}$), 7.13 (t, *J* = 6.3 Hz, 1H, $1'_{dU}$), 5.18 (q, *J* = 4.5 Hz, 1H, $3'_{dU}$), 4.56 (q, *J* = 2.8, 2.4 Hz, 1H, $4'_{dU}$), 4.33 (dd, *J* = 11.6, 2.9 Hz, 1H, $5'_{dU}$), 4.22 (dd, *J* = 11.8, 2.5 Hz, 1H, $5'_{dU}$), 2.85 (t, *J* = 5.8 Hz, 2H, $2'_{dU}$).

^{13}C NMR (126 MHz, Pyr-d_5): δ (ppm) = 163.3 ($CH_{arom.}$), 151.2 (C_q), 139.8 (C_q), 139.5 (C_q), 138.7 (6_{dU}), 133.7 (C_q), 133.0 (C_q), 131.5 ($3C_q$), 131.4 (C_q), 130.8 (C_q), 129.8 ($2CH_{arom.}$), 129.3

(C_q), 128.8 (C_q), 128.5 ($2CH_{arom.}$), 128.1 (C_q, $2CH_{arom.}$), 127.1 ($2CH_{arom.}$), 126.9 ($CH_{arom.}$), 126.2 (C_q), 120.9 ($2CH_{arom.}$), 120.7 ($CH_{arom.}$), 120.6 ($CH_{arom.}$), 114.1 (C_q), 88.8 ($4'_{dU}$), 85.8 ($1'_{dU}$), 70.9 ($3'_{dU}$), 61.6 ($5'_{dU}$), 41.9 ($2'_{dU}$).

HR-MS (ESI): m/z calculated for $C_{35}H_{26}O_5N_2^+$ [M^+] = 554.1842; found = 554.18292.

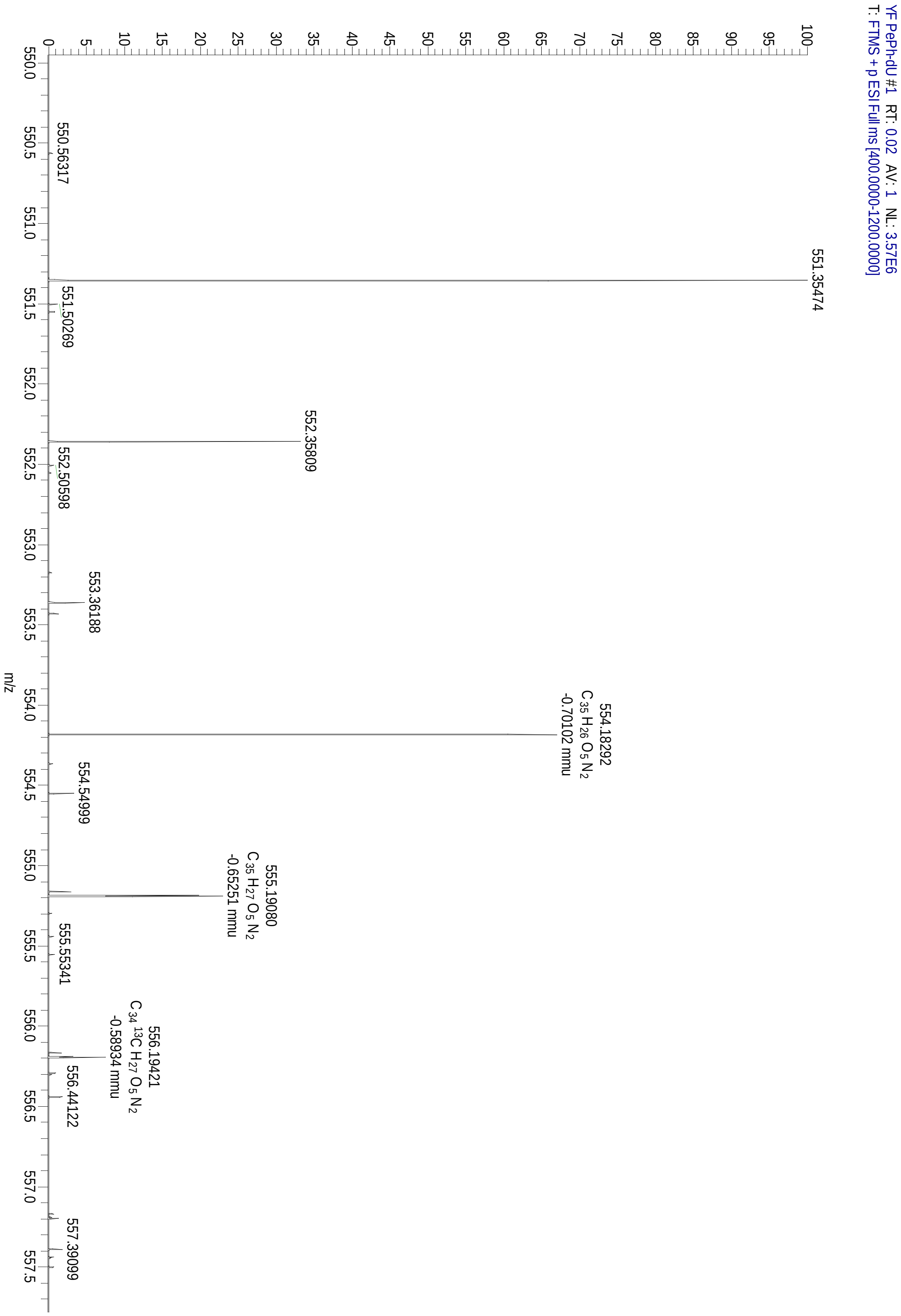
YF PePh-dU #1 RT: 0.02 AV: 1 NL: 3.57E6
T: FTMS + p ESI Full ms [400.0000-1200.0000]
551.35474
552.35809
554.18292
C_{35} H_{26} O_5 N_2
-0.70102 mmu
555.19080
C_{35} H_{27} O_5 N_2
-0.65251 mmu
556.19421
C_{34} ^{13}C H_{27} O_5 N_2
-0.58934 mmu
550.56317
551.50269
552.50598
553.36188
554.54999
555.55341
556.44122
557.39099
m/z

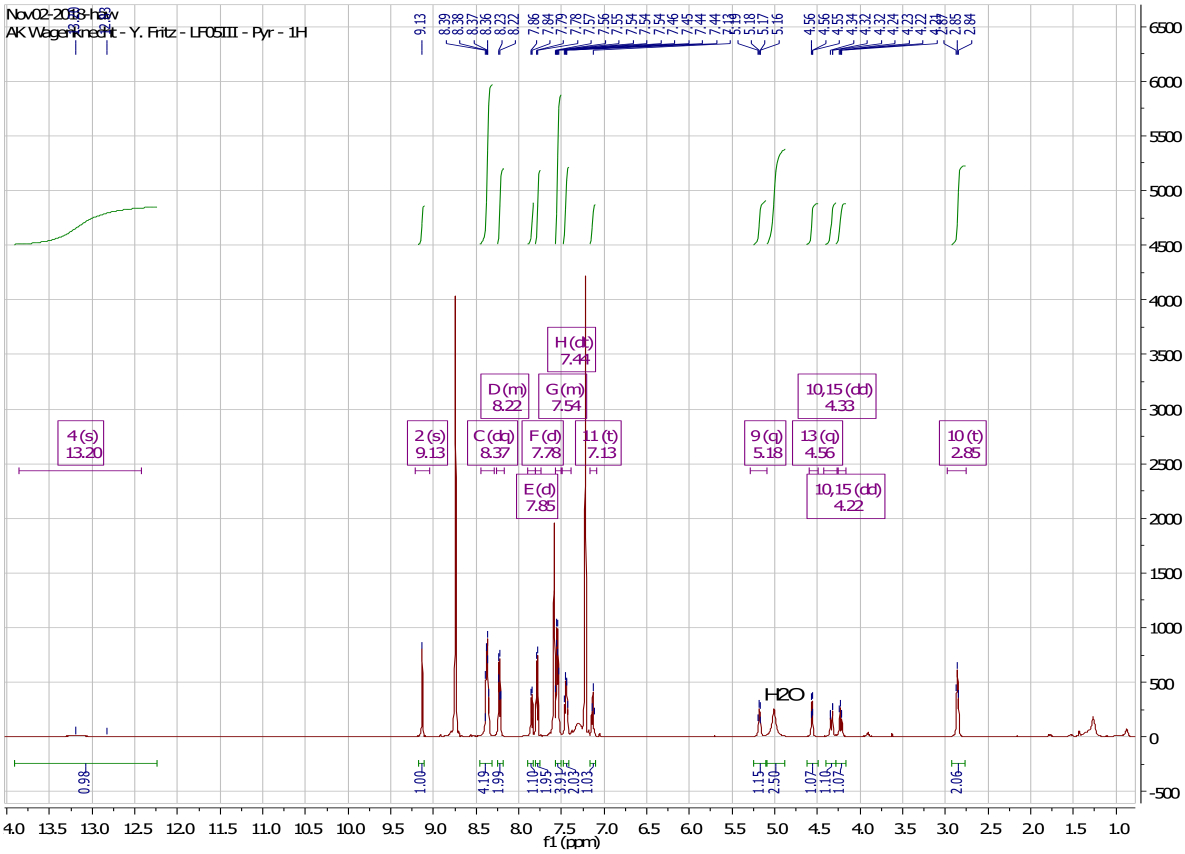
Nov02-2018-haw
AK Wagenknecht - Y. Fritz - LF05III - Pyr - 1H
4 (s) 13.20
2 (s) 9.13
C (dq) 8.37
D (m) 8.22
E (d) 7.85
F (d) 7.78
G (m) 7.54
H (dt) 7.44
11 (t) 7.13
9 (q) 5.18
13 (q) 4.56
10,15 (dd) 4.33
10,15 (dd) 4.22
10 (t) 2.85
H2O
0.98
1.00
4.19
1.99
1.10
1.95
3.91
2.03
1.03
1.15
2.50
1.07
1.10
1.07
2.06
f1 (ppm)

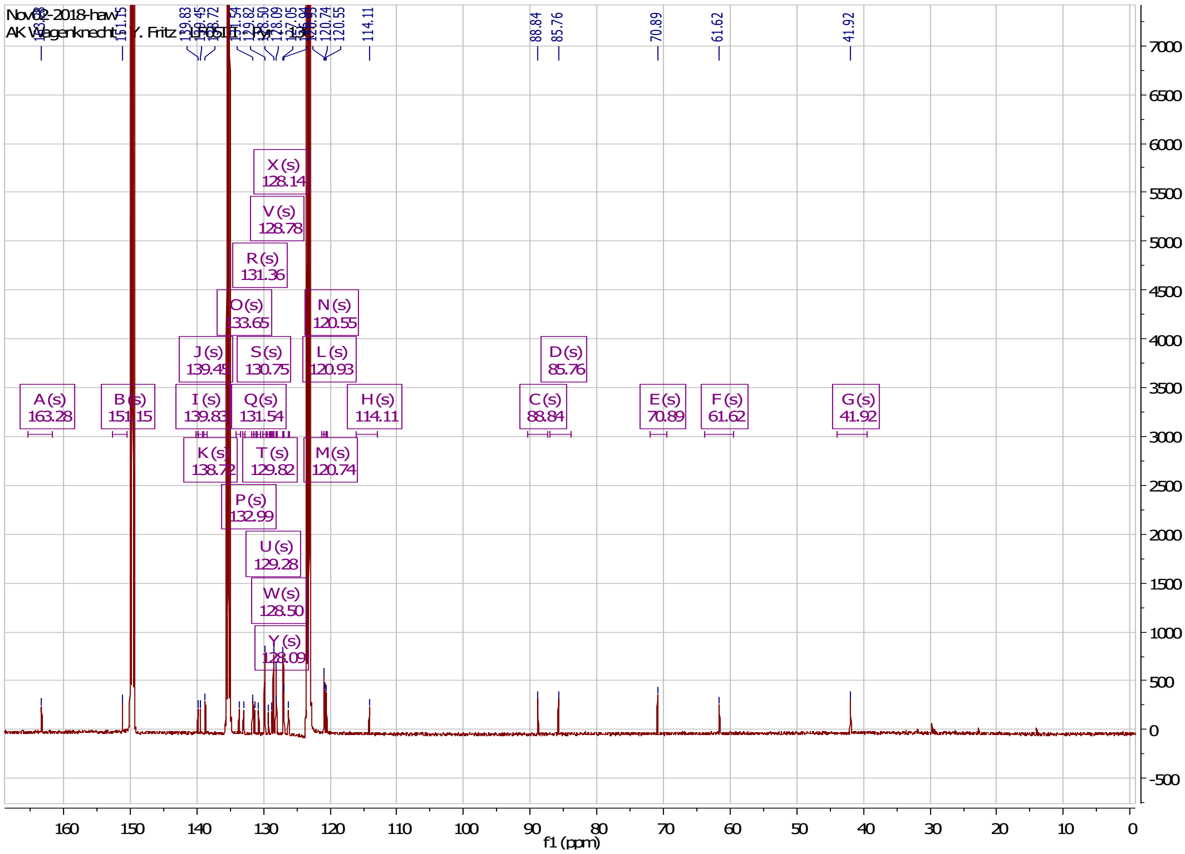
AK Wagenknecht
A (s) 163.28
I (s) 139.83
J (s) 139.45
K (s) 138.72
O (s) 133.65
P (s) 132.99
Q (s) 131.54
R (s) 131.36
S (s) 130.75
T (s) 129.82
U (s) 129.28
V (s) 128.78
W (s) 128.50
X (s) 128.14
Y (s) 128.09
L (s) 120.93
M (s) 120.74
N (s) 120.55
H (s) 114.11
C (s) 88.84
D (s) 85.76
E (s) 70.89
F (s) 61.62
G (s) 41.92
f1 (ppm)

2-Amino-8-(perylene-3-yl-*p*-phenyl)-2‘-deoxyadenosine (Pe-Ph-daA)

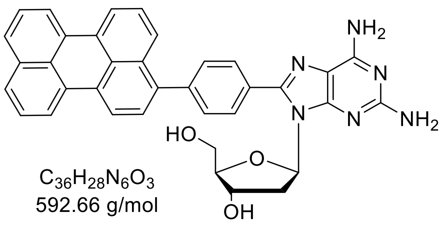

Pe-Ph-daA

The reaction was performed similar to **Pe-Ph-dU**. **9** was used instead of **6**. The crude product was purified by silica gel column chromatography (CH_2Cl_2:MeOH 50:1 – 10:1). The product was isolated as yellow solid with a yield of 29% (38 mg, 65.8 µmol).

TLC R_f(CH_2Cl_2:MeOH 10:1) = 0.39

^{1}H NMR (300 MHz, Pyr-d_5): δ (ppm) = 8.49 – 8.31 (m, 4$H_{arom.}$), 8.25 (s, 2H_{amino}), 8.20 – 8.05 (m, 2$H_{arom.}$), 7.80 (t, *J* = 8.4 Hz, 3$H_{arom.}$), 7.67 – 7.40 (m, 8$H_{arom.}$), 7.05 (dd, *J* = 9.2, 5.7 Hz, 1H, 1'$_{daA}$), 6.69 (s, 2H_{amino}), 5.26 (d, *J* = 5.1 Hz, 1H, 3'$_{daA}$), 4.69 (q, *J* = 2.5, 2.0 Hz, 1H, 4'$_{daA}$), 4.37 (dd, *J* = 12.3, 2.5 Hz, 1H, 5'$_{daA}$), 4.14 (d, *J* = 11.4 Hz, 1H, 5'$_{daA}$), 3.88 (ddd, *J* = 13.1, 9.5, 5.6 Hz, 1H, 2'$_{daA}$), 2.65 (dd, *J* = 12.8, 5.7 Hz, 1H, 2'$_{daA}$).

^{13}C NMR (126 MHz, Pyr-d_5): δ (ppm) = 161.4 ($CH_{arom.}$), 158.8 ($CH_{arom.}$), 153.6 (C_q), 148.4 (C_q), 142.4 (C_q), 139.8 (C_q), 133.5 (C_q), 132.3 (C_q), 132.2 (C_q), 132.0 (C_q), 131.8 (C_q), 131.0 (C_q, 2$CH_{arom.}$), 130.4 (2$CH_{arom.}$), 130.0 (C_q), 129.5 (C_q), 129.0 ($CH_{arom.}$), 128.9 (3C_q), 127.8 ($CH_{arom.}$), 127.8 (2$CH_{arom.}$), 126.7 ($CH_{arom.}$), 121.7 (2$CH_{arom.}$), 121.6 ($CH_{arom.}$), 121.2 ($CH_{arom.}$), 116.4 (C_q), 91.1 (4'$_{daA}$), 88.1 (1'$_{daA}$), 73.8 (3'$_{daA}$), 64.8 (5'$_{daA}$), 40.0 (2'$_{daA}$).

HR-MS (ESI): m/z calculated for $C_{36}H_{29}O_3N_6^+$ [M-H$^+$] = 593.2301; found = 593.22791.

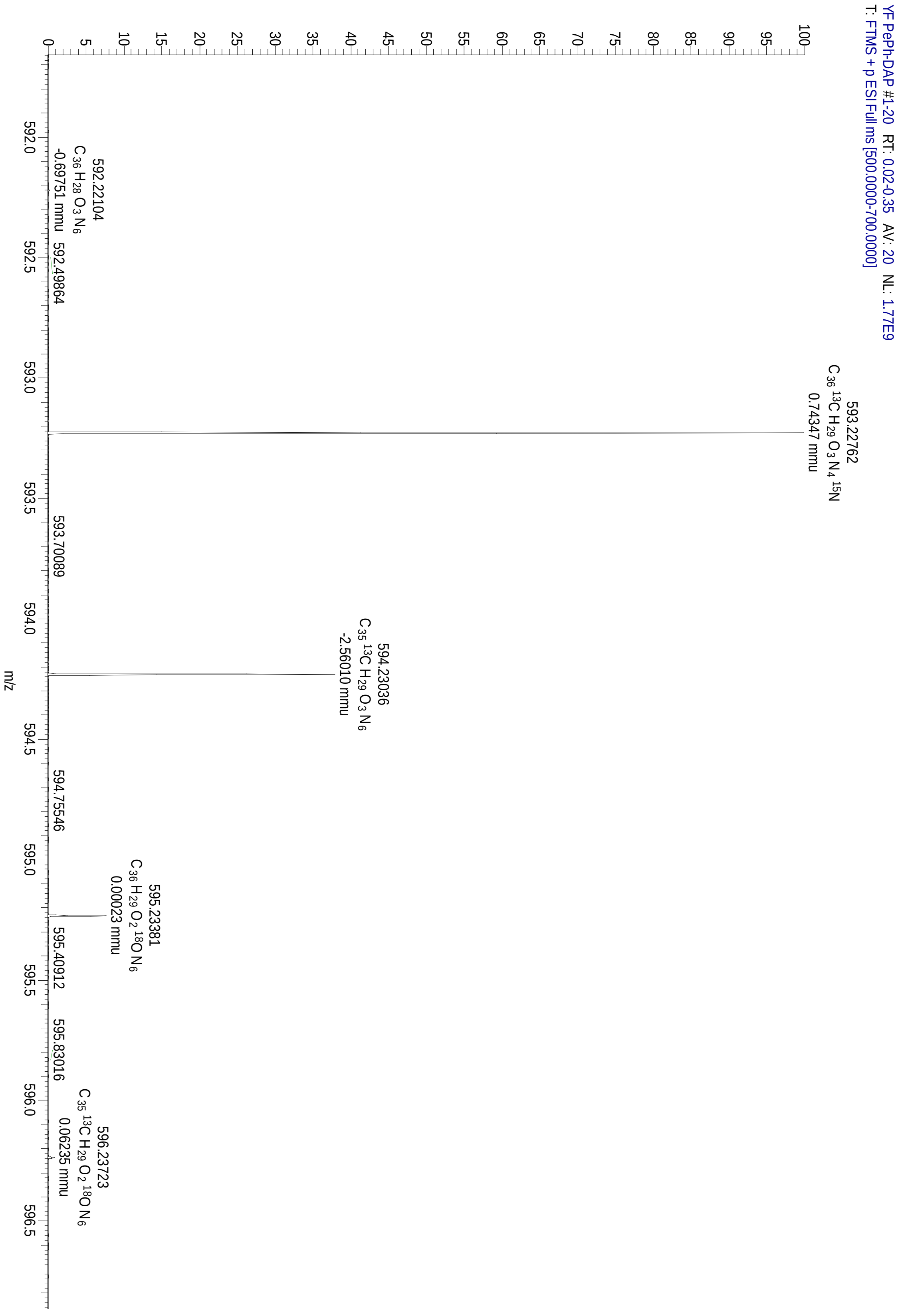

YF PePh-DAP #1-20 RT: 0.02-0.35 AV: 20 NL: 1.77E9
T: FTMS + p ESI Full ms [500.0000-700.0000]
593.22762
C36 13C H29 O3 N4 15N
0.74347 mmu
594.23036
C35 13C H29 O3 N6
-2.56010 mmu
595.23381
C36 H29 O2 18O N6
0.00023 mmu
596.23723
C35 13C H29 O2 18O N6
0.06235 mmu
592.22104
C36 H28 O3 N6
-0.69751 mmu
592.49864
593.70089
594.75546
595.40912
595.83016
m/z

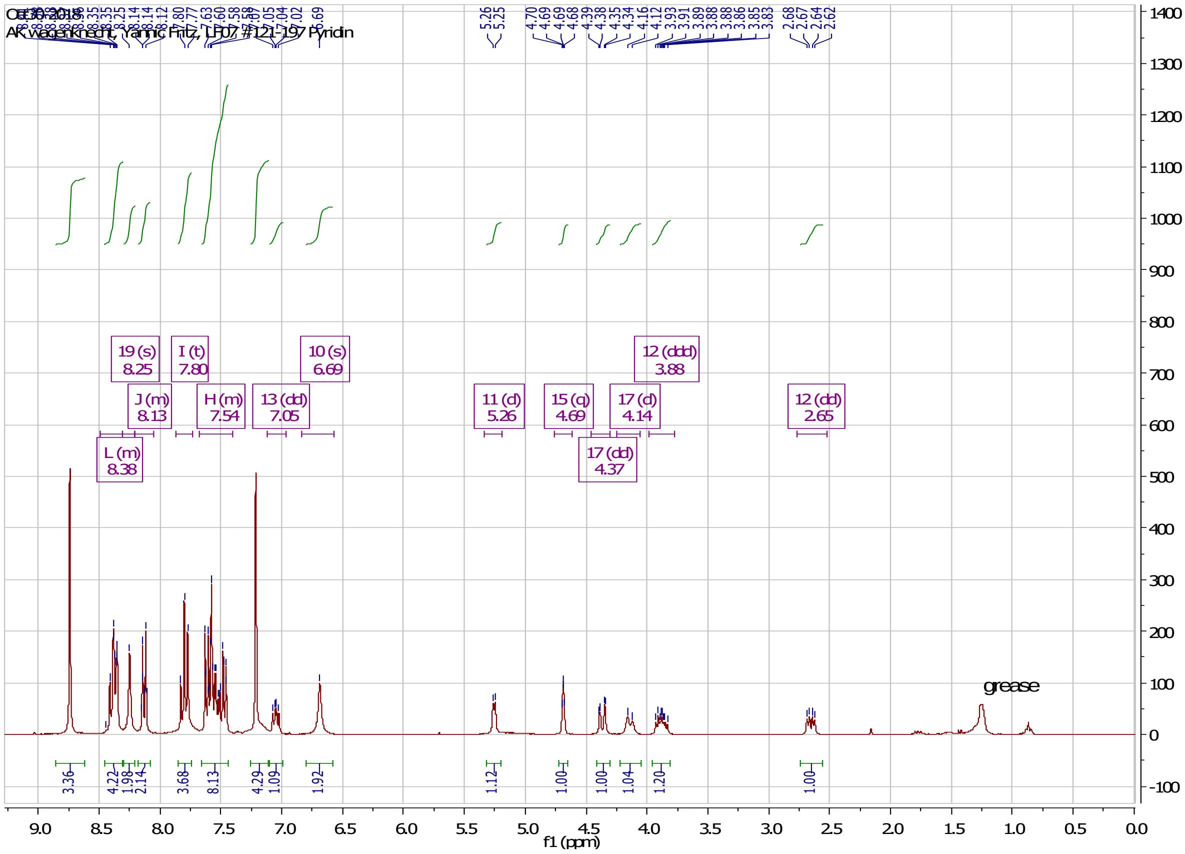

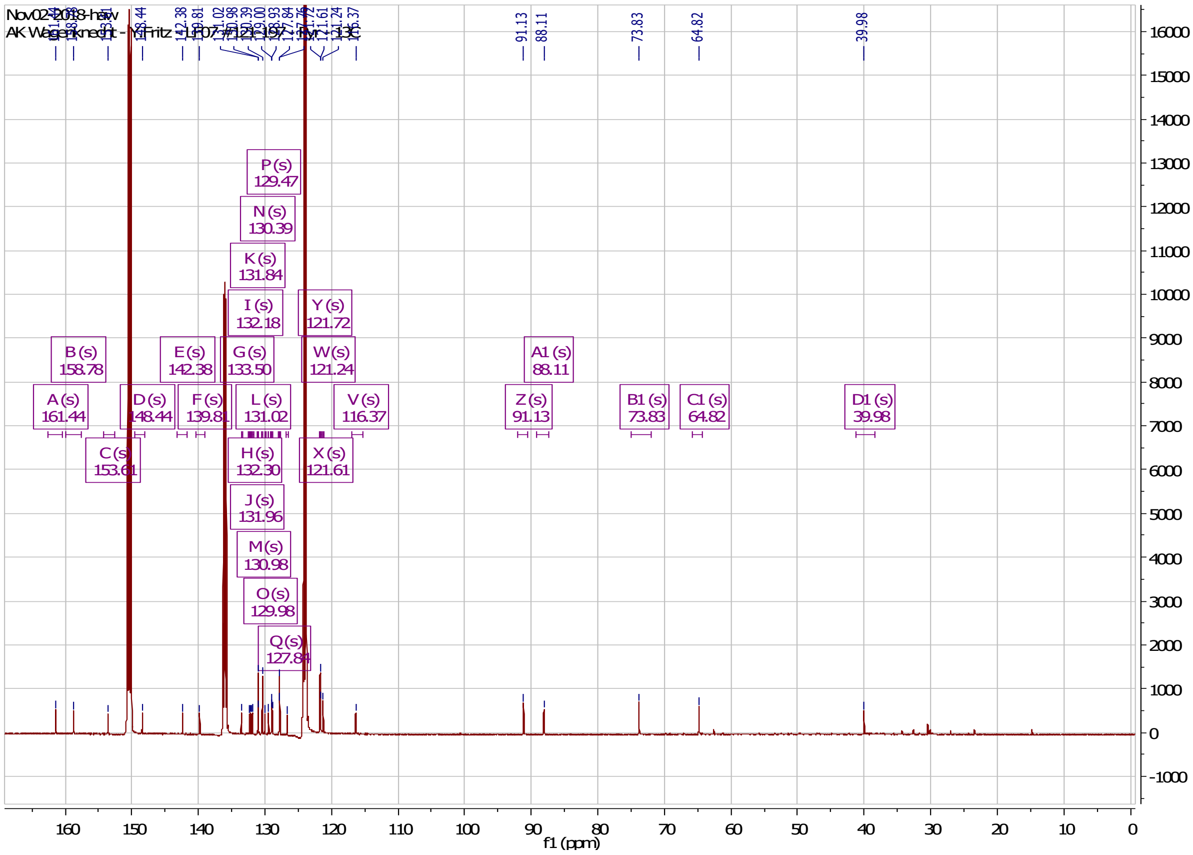

3-(*p*-Ethynylphenyl)-perylene (12)

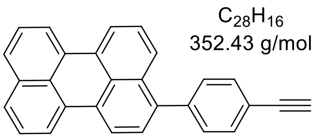

12

10 (195 mg, 553 µmol, 1.00 eq.), TMS-acetylene (490 µL, 337 mg, 3.43 mmol, 7.00 eq.) and CuI (2 mg, 10.5 µmol, 0.02 eq.) were suspended in NEt_3 (2 mL) and dioxane (2 mL). The suspension was degassed via freeze-pump-thaw and tetrakis(triphenylphosphine)palladium (26 mg, 22.5 µmol, 0.04 eq.) was added. The sealed vial was heated to 90 °C and stirred overnight. The solvent was removed under reduced pressure and filtrated through silica gel (CH_2Cl_2). The TMS-protected product was dissolved in THF (4 mL) and 1 M TBAF solution (740 µL) was added. The solution was stirred for 10 min at room temperature and the reaction was stopped by adding silica gel. The crude product was purified by silica gel column chromatography (hexane:CH_2Cl_2 1:1). The product was isolated as yellow solid with a yield of 77% (130 mg, 369 µmol).

TLC R_f(hexane:EtOAc 5:1) = 0.55 (TMS-product: 0.72)

^{1}H NMR (500 MHz, $CDCl_3$): δ (ppm) = 8.25 – 8.19 (m, 4H), 7.75 – 7.68 (m, 3H), 7.64 (d, *J* = 8.2 Hz, 2H), 7.53 – 7.48 (m, 4H), 7.44 (dd, *J* = 8.5, 7.5 Hz, 1H), 7.41 (d, *J* = 7.7 Hz, 1H), 3.16 (s, $1H_{acetylene}$).

^{13}C NMR (126 MHz, $CDCl_3$): δ (ppm) = 141.51 (C_q), 139.14 (C_q), 134.83 (C_q), 132.85 (C_q), 132.33 ($2CH_{arom.}$), 131.65 (C_q), 131.45 (C_q), 131.22 ($2C_q$), 130.09 ($2CH_{arom.}$), 129.23 (C_q), 128.77 (C_q), 128.07 ($2CH_{arom.}$), 127.84 ($CH_{arom.}$), 126.89 – 126.63 (C_q, $2CH_{arom.}$), 125.88 ($CH_{arom.}$), 121.28 (C_q), 120.59 ($2CH_{arom.}$), 120.41 ($CH_{arom.}$), 120.01 ($CH_{arom.}$), 83.71 ($C_{q,acetylene}$), 77.84 ($CH_{acetylene}$).

HR-MS (ESI): m/z calculated for $C_{28}H_{16}^{+}$ [M^{+}] = 352.1252; found = 352.12418.

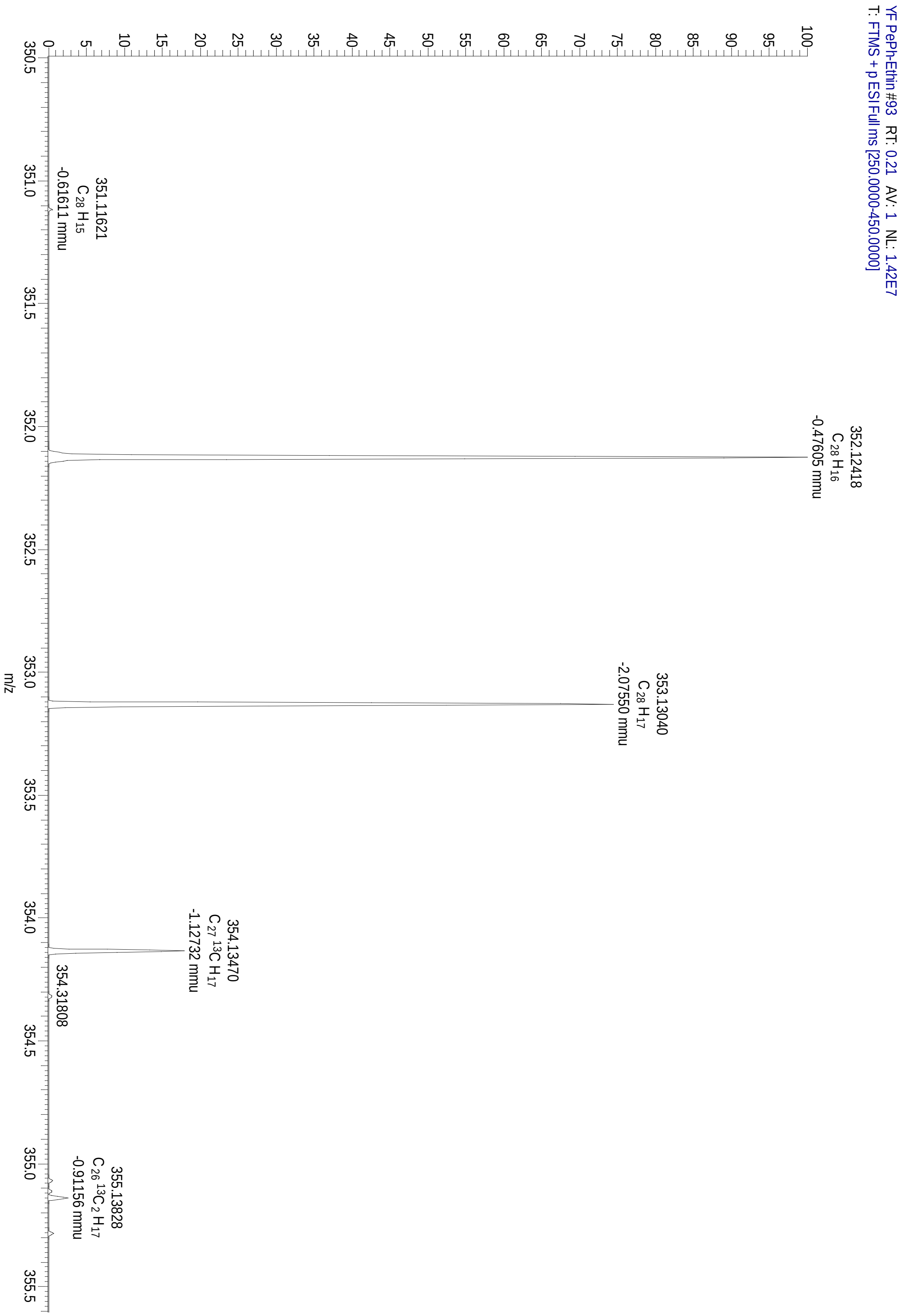

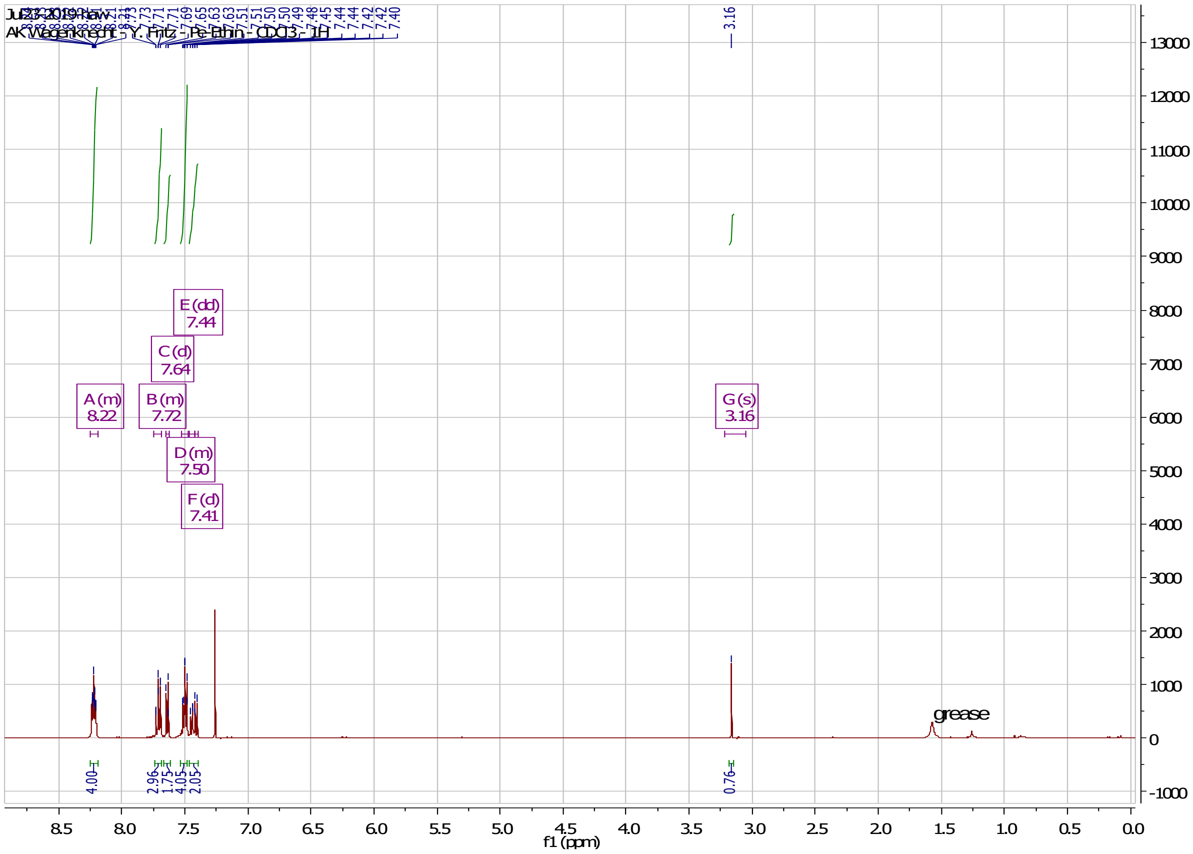
A (m) 8.22
B (m) 7.72
C (d) 7.64
D (m) 7.50
E (dd) 7.44
F (d) 7.41
G (s) 3.16
4.00
2.96
1.75
4.05
2.05
0.76
grease
f1 (ppm)

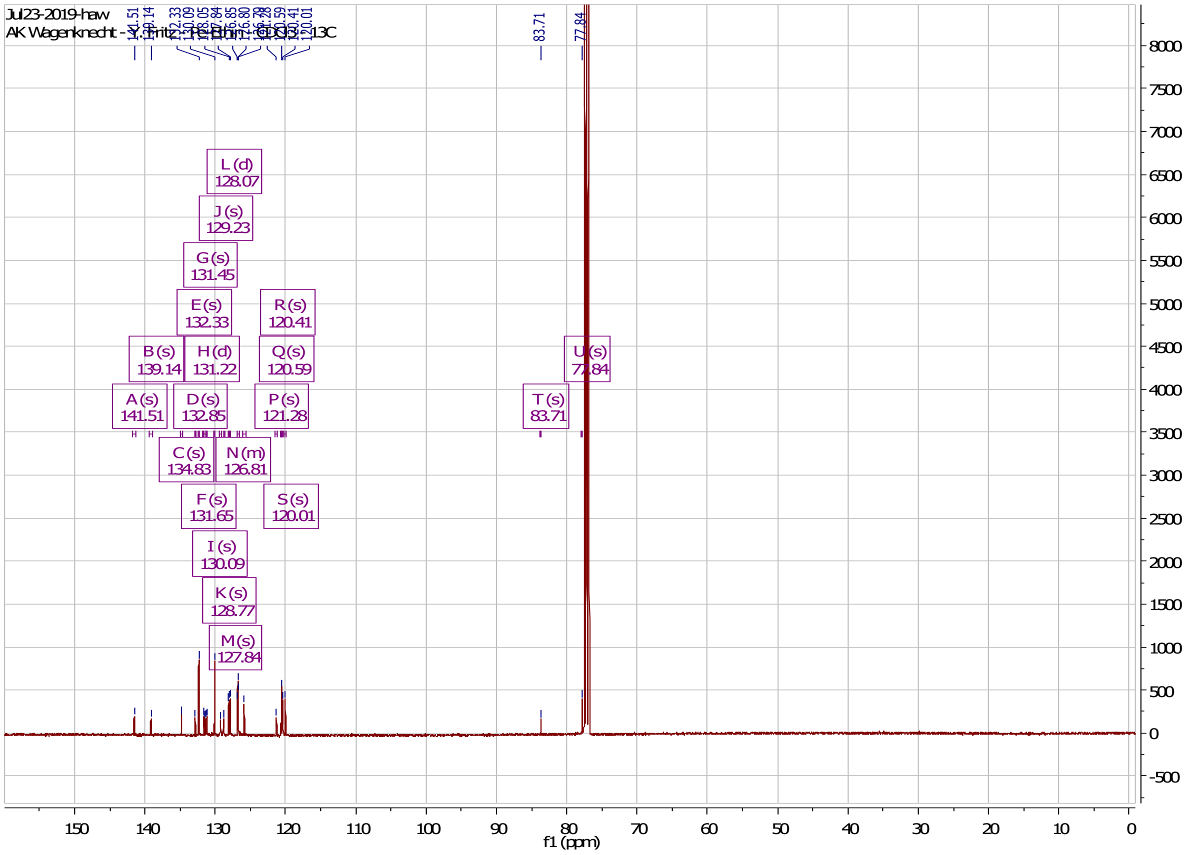
Jul23-2019-haw
A (s) 141.51
B (s) 139.14
C (s) 134.83
D (s) 132.85
E (s) 132.33
F (s) 131.65
G (s) 131.45
H (d) 131.22
I (s) 130.09
J (s) 129.23
K (s) 128.77
L (d) 128.07
M (s) 127.84
N (m) 126.81
P (s) 121.28
Q (s) 120.59
R (s) 120.41
S (s) 120.01
T (s) 83.71
U (s) 77.84
f1 (ppm)

5-(Perylene-3-yl(*p*-phenylethynyl))-2'-deoxyuridine (Pe-Ph-Et-dU)

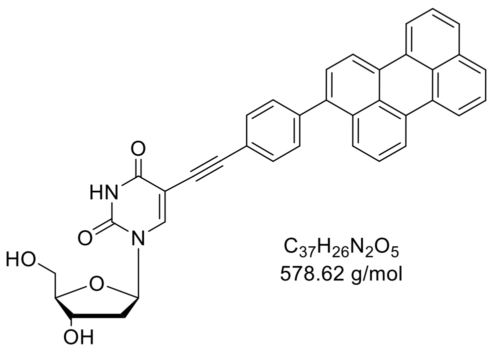

Pe-Ph-Et-dU

The reaction was performed similar to **Pe-Et-daA**. **7** and **12** were used instead of **5** and **9**. The crude product was purified by silica gel column chromatography (CH_2Cl_2:MeOH 50:1 – 15:1). The product was isolated as yellow solid with a yield of 55% (39 mg, 67.4 µmol).

TLC R_f(EtOAc) = 0.45

^{1}H NMR (300 MHz, Pyr-d_5): δ (ppm) = 9.25 (s, 1H, 6_{dU}), 8.38 (dd, *J* = 7.5, 4.7 Hz, $4H_{arom.}$), 7.80 (t, *J* = 7.8 Hz, $3H_{arom.}$), 7.68 – 7.61 (m, $2H_{arom.}$), 7.61 – 7.50 (m, $3H_{arom.}$), 7.46 (q, *J* = 7.5, 6.9 Hz, $3H_{arom.}$), 6.99 (t, *J* = 6.3 Hz, 1H, $1'_{dU}$), 5.18 – 5.07 (m, 1H, $3'_{dU}$), 4.54 (q, *J* = 3.0 Hz, 1H, $4'_{dU}$), 4.33 (dd, *J* = 11.8, 2.9 Hz, 1H, $5'_{dU}$), 4.21 (dd, *J* = 11.7, 2.7 Hz, 1H, $5'_{dU}$), 2.77 (dq, *J* = 13.0, 5.7 Hz, 2H, $2'_{dU}$).

^{13}C NMR (126 MHz, Pyr-d_5): δ (ppm) = 163.3 ($CH_{arom.}$), 151.4 ($CH_{arom.}$), 145.1 ($CH_{arom.}$), 141.3 (C_q), 139.9 (C_q), 133.5 (C_q), 132.4 (C_q, $2CH_{arom.}$), 132.2 (C_q), 132.0 (C_q), 131.8 (C_q), 130.8 ($2CH_{arom.}$), 130.0 (C_q), 129.5 (C_q), 128.9 ($CH_{arom.}$), 128.8 ($CH_{arom.}$), 127.8 ($3CH_{arom.}$), 126.6 (C_q), 121.7 ($2CH_{arom.}$), 121.6 ($CH_{arom.}$), 121.2 ($CH_{arom.}$), 100.4 (C_q), 93.2 (C_q), 89.7 ($4'_{dU}$), 86.8 ($1'_{dU}$), 84.8 (C_q), 71.5 ($3'_{dU}$), 71.3 (C_q), 62.3 ($5'_{dU}$), 42.6 ($2'_{dU}$).

HR-MS (ESI): m/z calculated for $C_{37}H_{26}O_5N_2^+$ [M^+] = 578.1842; found = 578.18164.

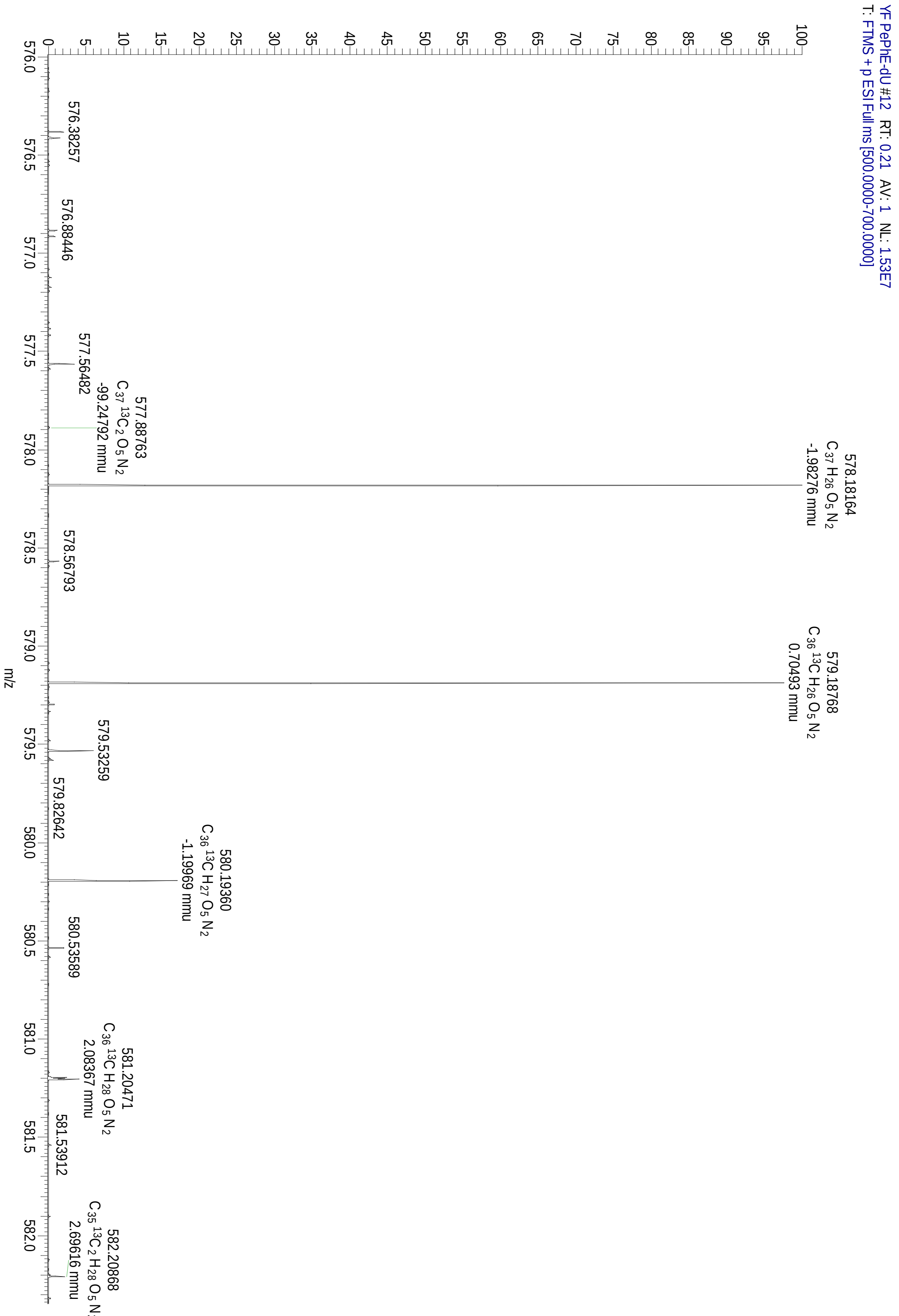
YF PePhE-dU #12 RT: 0.21 AV: 1 NL: 1.53E7
T: FTMS + p ESI Full ms [500.0000-700.0000]
578.18164
C37 H26 O5 N2
-1.98276 mmu
579.18768
C36 13C H26 O5 N2
0.70493 mmu
580.19360
C36 13C H27 O5 N2
-1.19969 mmu
581.20471
C36 13C H28 O5 N2
2.08367 mmu
582.20868
C35 13C2 H28 O5 N2
2.69616 mmu
577.88763
C37 13C2 O5 N2
-99.24792 mmu
576.38257
576.88446
577.56482
578.56793
579.53259
579.82642
580.53589
581.53912
m/z

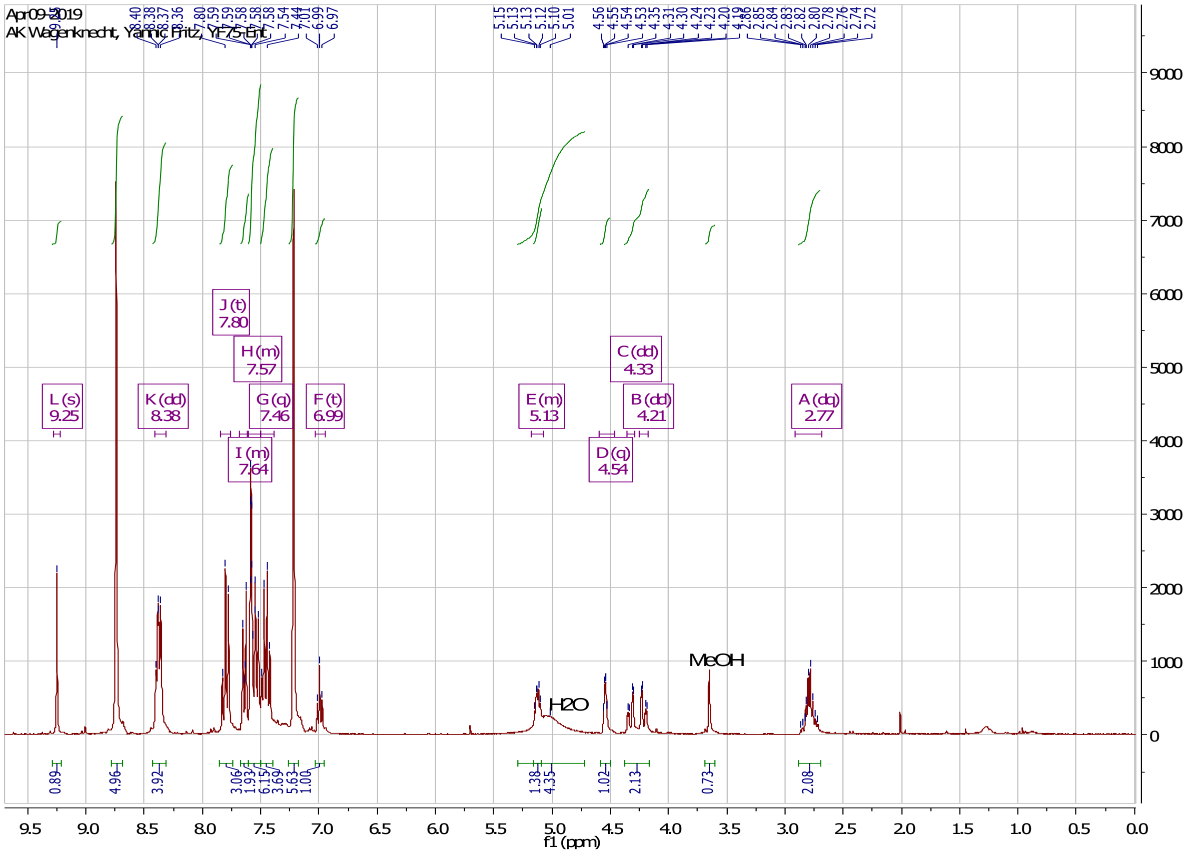
Apr09-2019
AK Wagenknecht, Yannic Fritz, YF75 Ent
L (s) 9.25
K (dd) 8.38
J (t) 7.80
H (m) 7.57
I (m) 7.64
G (q) 7.46
F (t) 6.99
E (m) 5.13
C (dd) 4.33
D (q) 4.54
B (dd) 4.21
A (dq) 2.77
H2O
MeOH
f1 (ppm)

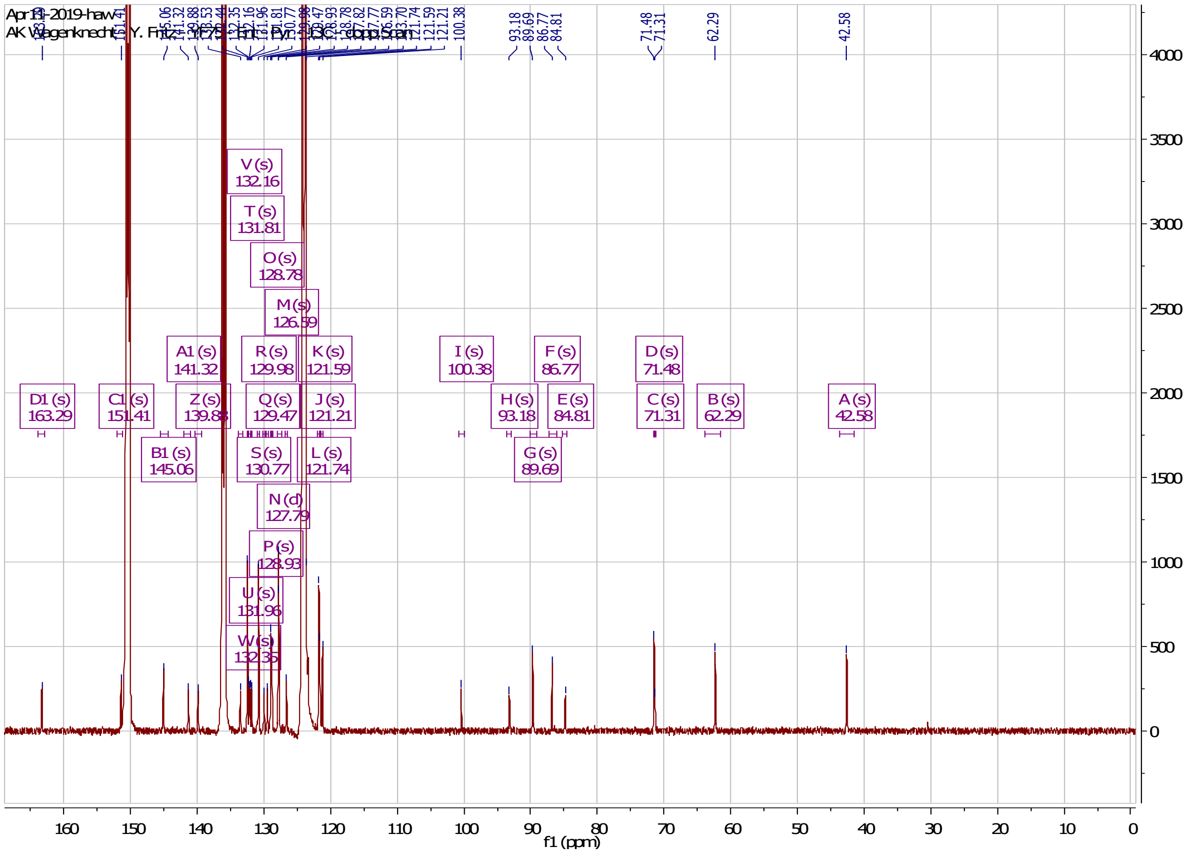
f1 (ppm)
D1 (s) 163.29
C1 (s) 151.41
B1 (s) 145.06
A1 (s) 141.32
Z (s) 139.88
V (s) 132.16
T (s) 131.81
O (s) 128.78
M (s) 126.59
R (s) 129.98
K (s) 121.59
Q (s) 129.47
J (s) 121.21
S (s) 130.77
L (s) 121.74
N (d) 127.79
P (s) 128.93
U (s) 131.96
W (s) 132.35
I (s) 100.38
H (s) 93.18
F (s) 86.77
E (s) 84.81
G (s) 89.69
D (s) 71.48
C (s) 71.31
B (s) 62.29
A (s) 42.58

2-Amino-8-(perylene-3-yl(*p*-phenylethynyl))-2'-deoxyadenosine (Pe-Ph-Et-daA)

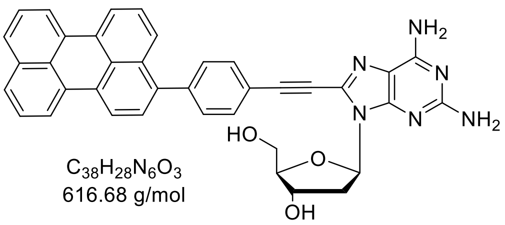

$C_{38}H_{28}N_6O_3$
616.68 g/mol

Pe-Ph-Et-daA

The reaction was performed similar to **Pe-Et-daA**. **12** was used instead of **5**. The product was isolated as yellow solid with a yield of 77% (85 mg, 138 µmol).

TLC $R_f(CH_2Cl_2$:MeOH 10:1) = 0.43

^{1}H NMR (300 MHz, Pyr-d_5): δ (ppm) = 8.38 (m, Hz, 4$H_{arom.}$, 2H_{amino}), 7.81 (dd, *J* = 8.4, 4.8 Hz, 3$H_{arom.}$), 7.66 – 7.51 (m, 5$H_{arom.}$), 7.46 (dd, *J* = 8.0, 4.5 Hz, 3$H_{arom.}$), 7.31 (dd, *J* = 9.0, 5.9 Hz, 1H, 1'$_{daA}$), 6.90 (s, 2H_{amino}), 5.26 (d, *J* = 5.1 Hz, 1H, 3'$_{daA}$), 4.74 (s, 1H, 4'$_{daA}$), 4.35 (dd, *J* = 12.2, 2.6 Hz, 1H, 5'$_{daA}$), 4.13 (dd, *J* = 12.4, 2.9 Hz, 1H, 5'$_{daA}$), 3.76 – 3.68 (m, 1H, 2'$_{daA}$), 2.69 (dd, *J* = 12.8, 6.0 Hz, 1H, 2'$_{daA}$).

^{13}C NMR (126 MHz, Pyr-d_5): δ (ppm) = 162.4 ($CH_{arom.}$), 158.7 ($CH_{arom.}$), 152.1 ($CH_{arom.}$), 142.6 (C_q), 139.6 (C_q), 133.4 (C_q), 132.8 (2$CH_{arom.}$), 132.4 (C_q), 132.1 (C_q), 132.0 (2C_q), 131.6 (C_q), 130.9 (2$CH_{arom.}$), 130.0 (C_q), 129.5 (C_q), 129.0 (3C_q), 128.8 ($CH_{arom.}$), 127.9 ($CH_{arom.}$), 127.8 (2$CH_{arom.}$), 126.5 (C_q), 121.8 (2$CH_{arom.}$), 121.7 ($CH_{arom.}$), 121.2 (C_q, $CH_{arom.}$), 116.9 (C_q), 94.6 (C_q), 91.2 (3'$_{daA}$), 87.9 (1'$_{daA}$), 81.4 (C_q), 73.8 (4'$_{daA}$), 64.7 (5'$_{daA}$), 40.4 (2'$_{daA}$).

HR-MS (ESI): m/z calculated for $C_{38}H_{29}O_3N_6^+$ [M-H^+] = 617.2301; found = 617.22870.

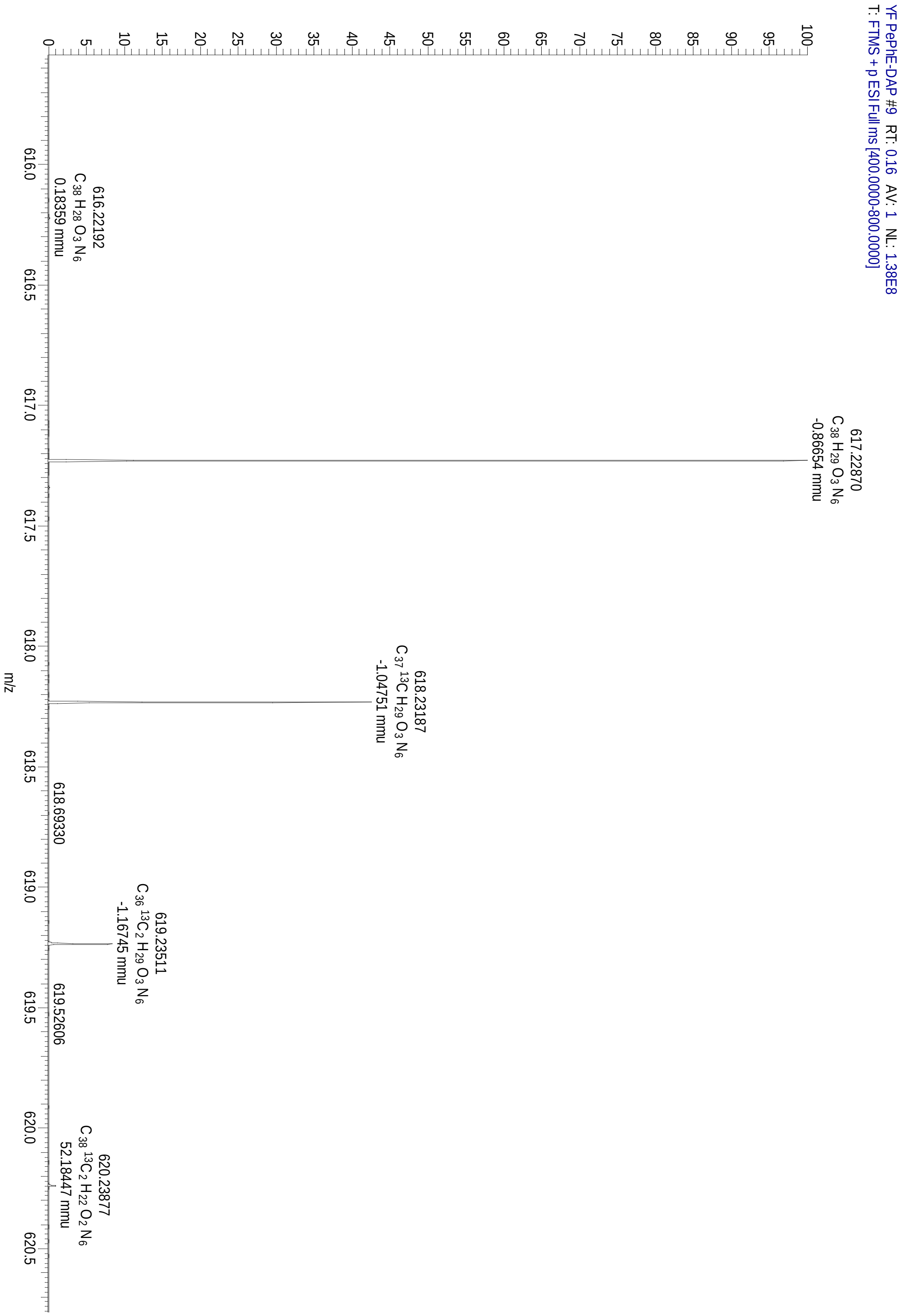
YF PePhE-DAP #9 RT: 0.16 AV: 1 NL: 1.38E8
T: FTMS + p ESI Full ms [400.0000-800.0000]
616.22192
C38 H28 O3 N6
0.18359 mmu
617.22870
C38 H29 O3 N6
-0.86654 mmu
618.23187
C37 13C H29 O3 N6
-1.04751 mmu
618.69330
619.23511
C36 13C2 H29 O3 N6
-1.16745 mmu
619.52606
620.23877
C38 13C2 H22 O2 N6
52.18447 mmu
m/z

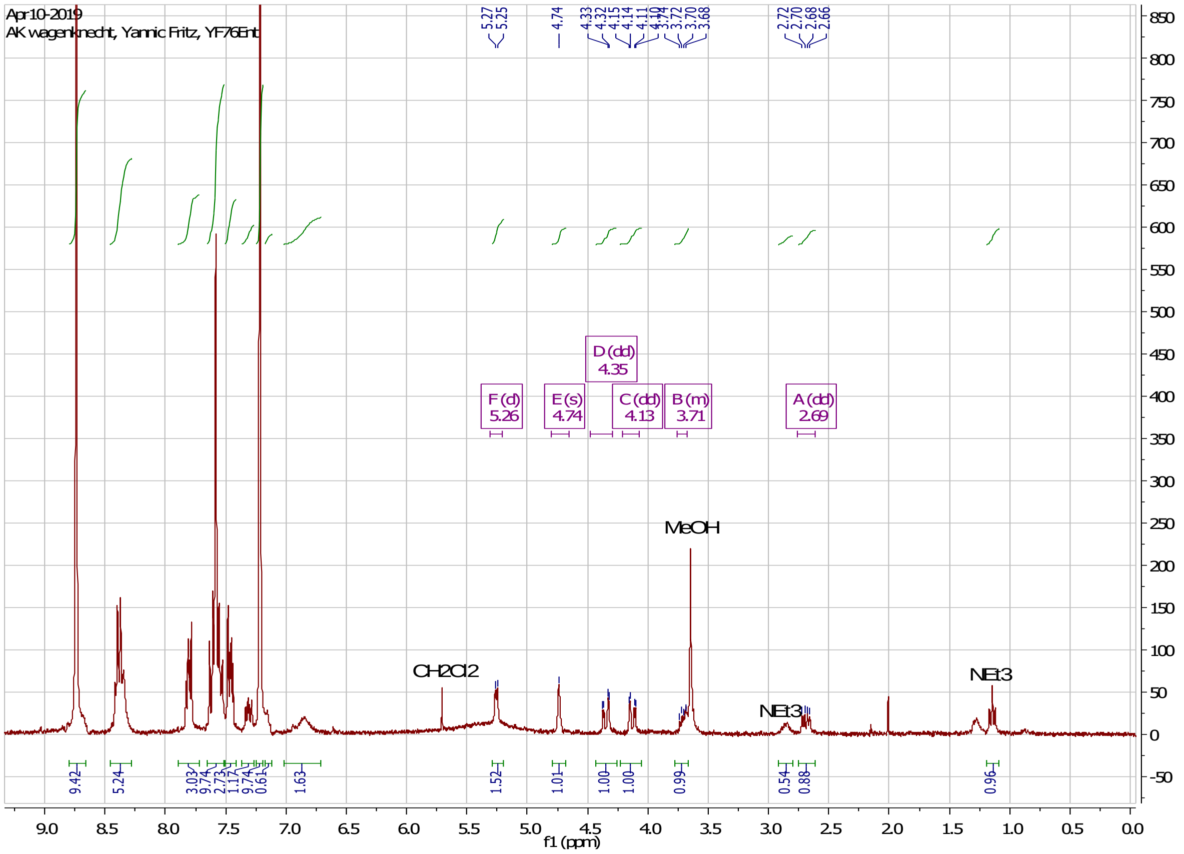

Apr10-2019
AK wagenknecht, Yannic Fritz, YF76Ent
MeOH
CH2Cl2
NEt3
NEt3
f1 (ppm)

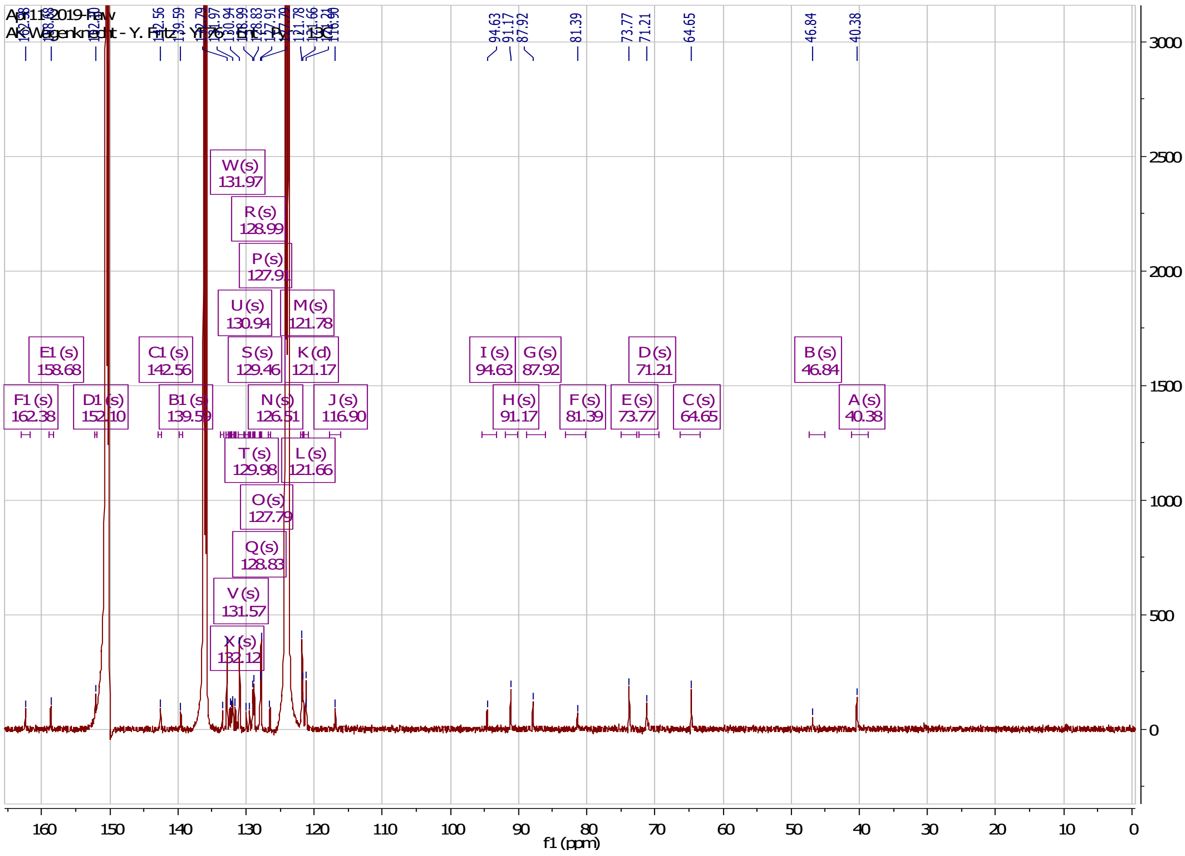

f1 (ppm)

Additional Spectra

Emission spectra

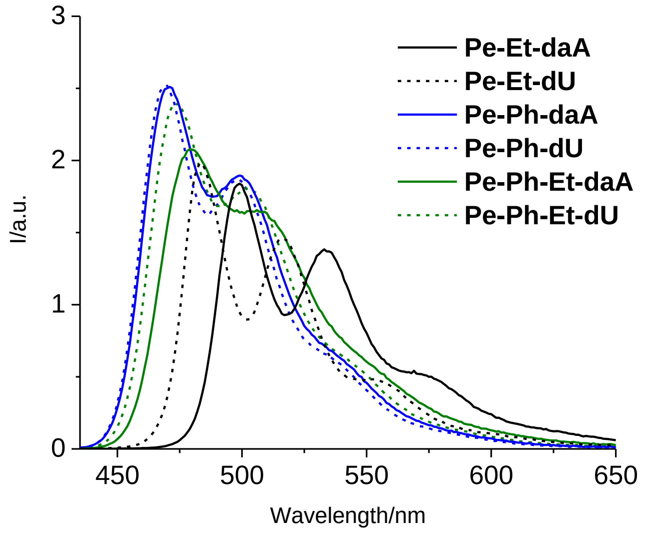

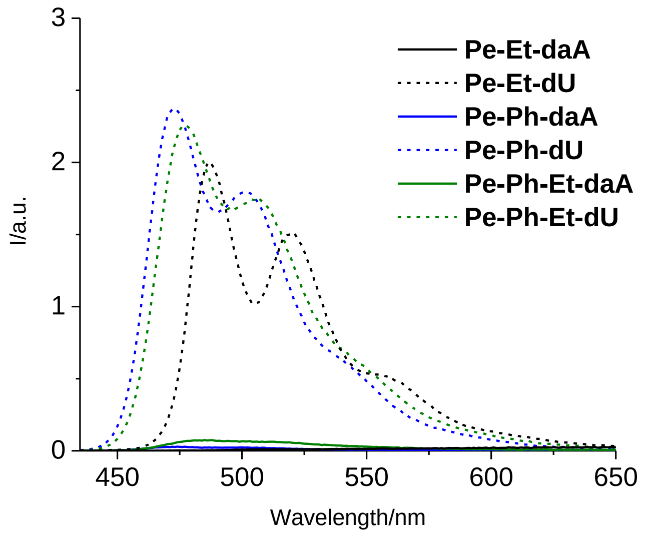

Figure S1: Emission spectra of all perylene-conjugates in chloroform with 2% DMSO (top) and DMSO (bottom), each 20 µM. Excitation at 420 nm.

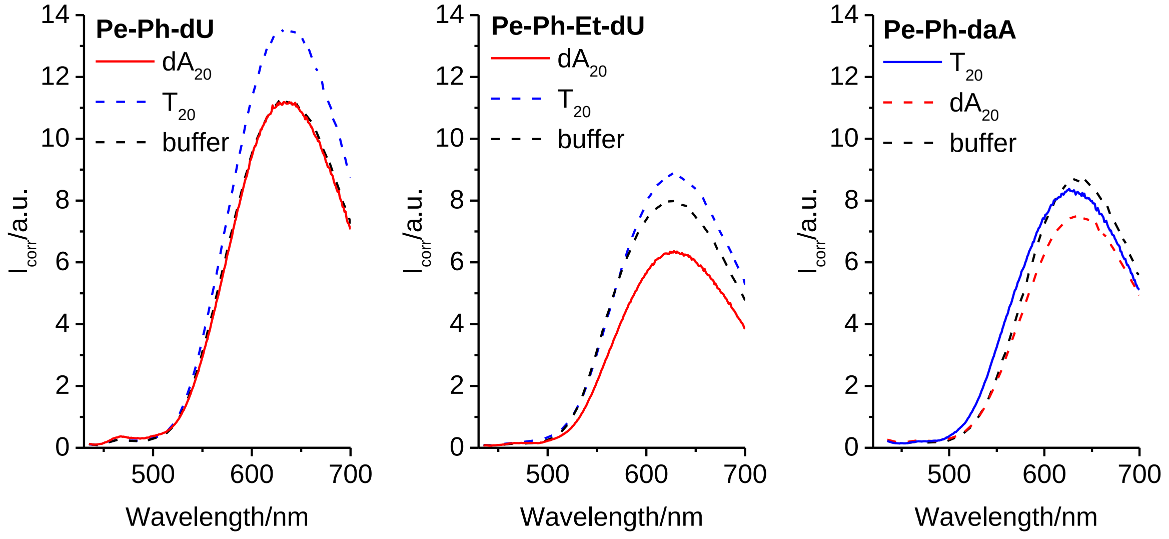

Figure S2: Emission spectra of **Pe-Ph-dU** (left), **Pe-Ph-Et-dU** (middle) and **Pe-Ph-daA** (right), each 45 µM in aqueous buffer (10 mM Na-Pi, 250 mM NaCl, with 4.5% DMSO) without and with DNA templates $\mathbf{T_{20}}$ and $\mathbf{dA_{20}}$ (1,5 µM), respectively. Excitation at 420 nm and divided by absorbance at 420 nm.

UV/vis-absorption spectra

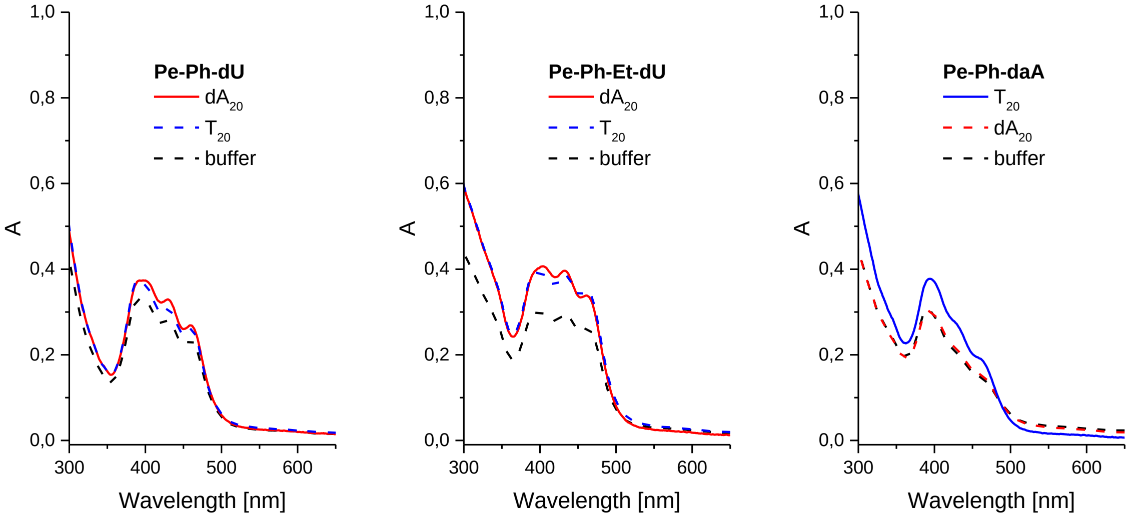

Figure S3: UV/vis absorbance of **Pe-Ph-dU** (left), **Pe-Ph-Et-dU** (middle) and **Pe-Ph-daA** (right), each 45 µM in aqueous buffer (10 mM Na-Pi, 250 mM NaCl, with 4.5% DMSO) without and with DNA templates $\mathbf{T_{20}}$ and $\mathbf{dA_{20}}$ (1,5 µM), respectively.

CD-spectra

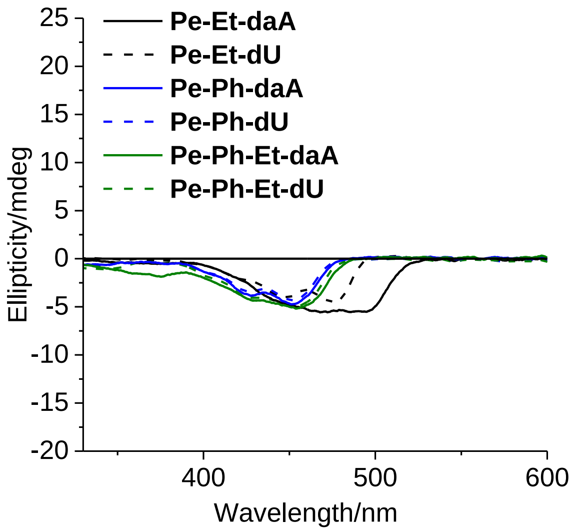

Figure S4. CD of all perylene-conjugates in DMSO (20 µM).

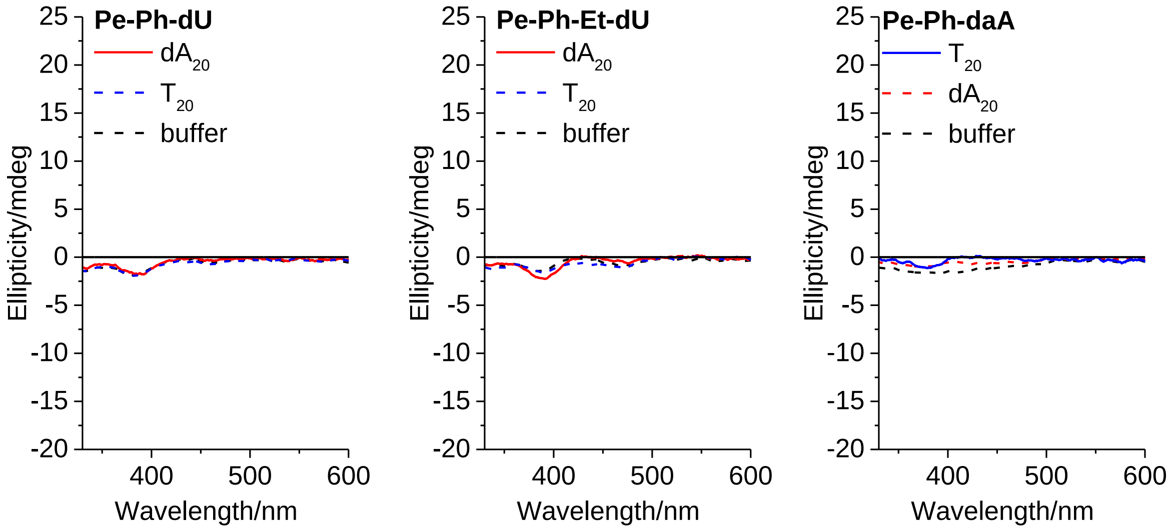

Figure S5. CD of **Pe-Ph-dU** (left), **Pe-Ph-Et-dU** (middle) and **Pe-Ph-daA** (right), each 45 µM in aqueous buffer (10 mM Na-Pi, 250 mM NaCl, with 4.5% DMSO) without and with DNA templates **T_{20}** and **dA_{20}** (1,5 µM), respectively.

References

Andronova, V.L., Skorobogatyi, M.V., Manasova, E.V., Berlin, Y.A., Korshun, V.A., and Galegov, G.A. (2003). Antiviral Activity of Some 2'-Deoxyuridine 5-Arylethynyl Derivatives. *Russian Journal of Bioorganic Chemistry* 29(3), 262-266. doi: 10.1023/a:1023936516589.

Castanet, A.-S., Colobert, F., and Broutin, P.-E. (2002). Mild and regioselective iodination of electron-rich aromatics with N -iodosuccinimide and catalytic trifluoroacetic acid. *Tetrahedron Letters* 43(29), 5047-5048. doi: 10.1016/s0040-4039(02)01010-9.

Hayashi, K., and Inouye, M. (2017). Reliable and Reproducible Separation of 3,9- and 3,10-Dibromoperylenes and the Photophysical Properties of Their Alkynyl Derivatives. *European Journal of Organic Chemistry* 2017(29), 4334-4337. doi: 10.1002/ejoc.201700807.

Okamoto, A., Ochi, Y., and Saito, I. (2005). Fluorometric sensing of the salt-induced B–Z DNA transition by combination of two pyrene-labeled nucleobases. *Chemical Communications* (9), 1128-1130. doi: 10.1039/B416965D.

Okamoto, S., Kojiyama, K., Tsujioka, H., and Sudo, A. (2016). Metal-free reductive coupling of C[double bond, length as m-dash]O and C[double bond, length as m-dash]N bonds driven by visible light: use of perylene as a simple photoredox catalyst. *Chemical Communications* 52(76), 11339-11342. doi: 10.1039/c6cc05867a.

Riedl, J., Pohl, R., Rulisek, L., and Hocek, M. (2012). Synthesis and photophysical properties of biaryl-substituted nucleos(t)ides. Polymerase synthesis of DNA probes bearing solvatochromic and pH-sensitive dual fluorescent and 19F NMR labels. *Journal of Organic Chemistry* 77(2), 1026-1044. doi: 10.1021/jo202321g.

Yamaji, M., Maeda, H., Nanai, Y., and Mizuno, K. (2012). Substitution effects of CC triple bonds on the fluorescent properties of perylenes studied by emission and transient absorption measurements. *Chemical Physics Letters* 536, 72-76. doi: 10.1016/j.cplett.2012.03.108.
